# Scaffold protein SH3BP2 signalosome is pivotal for immune activation in nephrotic syndrome

**DOI:** 10.1172/jci.insight.170055

**Published:** 2024-02-08

**Authors:** Tarak Srivastava, Robert E. Garola, Jianping Zhou, Varun C. Boinpelly, Mohammad H. Rezaiekhaligh, Trupti Joshi, Yuexu Jiang, Diba Ebadi, Siddarth Sharma, Christine Sethna, Vincent S. Staggs, Ram Sharma, Debbie S. Gipson, Wei Hao, Yujie Wang, Laura H. Mariani, Jeffrey B. Hodgin, Robert Rottapel, Teruhito Yoshitaka, Yasuyoshi Ueki, Mukut Sharma

**Affiliations:** 1 Section of Nephrology, Children’s Mercy Hospital and University of Missouri at Kansas City, Kansas City, Missouri, USA.; 2 Midwest Veterans’ Biomedical Research Foundation, Kansas City, Missouri, USA.; 3 Department of Oral and Craniofacial Sciences, University of Missouri at Kansas City School of Dentistry, Kansas City, Missouri, USA.; 4 Department of Pathology and Laboratory Medicine, Children’s Mercy Hospital and University of Missouri at Kansas City, Kansas City, Missouri, USA.; 5 Kansas City VA Medical Center, Kansas City, Missouri, USA.; 6 Department of Health Management and Informatics,; 7 Department of Electrical Engineering and Computer Science,; 8 Christopher S. Bond Life Sciences Center, and; 9 MU Institute for Data Science and Informatics, University of Missouri, Columbia, Missouri, USA.; 10 The Ottawa Hospital Rehabilitation Centre, Ottawa, Ontario, Canada.; 11 Milken Institute School of Public Health, George Washington University, Washington, DC, USA.; 12 Cohen Children’s Medical Center of NY, New Hyde Park, New York, USA.; 13 Biostatistics and Epidemiology Core, Children’s Mercy Research Institute and Department of Pediatrics, University of Missouri, Kansas City, Missouri, USA.; 14 Department of Internal Medicine, The Jared Grantham Kidney Institute, University of Kansas Medical Center, Kansas City, Kansas, USA.; 15 Division of Nephrology, Department of Internal Medicine, School of Medicine, and; 16 Department of Biostatistics, School of Public Health, University of Michigan, Ann Arbor, Michigan, USA.; 17 Princess Margaret Cancer Center, University Health Network, University of Toronto, Toronto, Ontario, Canada.; 18 Department of Orthopedic Surgery, Hiroshima City Rehabilitation Hospital, Hiroshima, Hiroshima, Japan.; 19 Department of Biomedical Sciences and Comprehensive Care, Indiana University School of Dentistry, Indianapolis, Indiana, USA.; 20 Indiana Center for Musculoskeletal Health, Indiana University School of Medicine, Indianapolis, Indiana, USA.

**Keywords:** Nephrology, Innate immunity

## Abstract

Despite clinical use of immunosuppressive agents, the immunopathogenesis of minimal change disease (MCD) and focal segmental glomerulosclerosis (FSGS) remains unclear. Src homology 3-binding protein 2 (SH3BP2), a scaffold protein, forms an immune signaling complex (signalosome) with 17 other proteins, including phospholipase Cγ2 (PLCγ2) and Rho-guanine nucleotide exchange factor VAV2 (VAV2). Bioinformatic analysis of human glomerular transcriptome (Nephrotic Syndrome Study Network cohort) revealed upregulated *SH3BP2* in MCD and FSGS. The SH3BP2 signalosome score and downstream *MyD88*, *TRIF*, and *NFATc1* were significantly upregulated in MCD and FSGS. Immune pathway activation scores for Toll-like receptors, cytokine-cytokine receptor, and NOD-like receptors were increased in FSGS. Lower SH3BP2 signalosome score was associated with MCD, higher estimated glomerular filtration rate, and remission. Further work using *Sh3bp2^KI/KI^* transgenic mice with a gain-in-function mutation showed ~6-fold and ~25-fold increases in albuminuria at 4 and 12 weeks, respectively. Decreased serum albumin and unchanged serum creatinine were observed at 12 weeks. *Sh3bp2^KI/KI^* kidney morphology appeared normal except for increased mesangial cellularity and patchy foot process fusion without electron-dense deposits. SH3BP2 co-immunoprecipitated with PLCγ2 and VAV2 in human podocytes, underscoring the importance of SH3BP2 in immune activation. SH3BP2 and its binding partners may determine the immune activation pathways resulting in podocyte injury leading to loss of the glomerular filtration barrier.

## Abstract 2

<p>Results from human glomerular transcriptome and from gain-of-function *Sh3bp2^KI/KI^* mice suggest role for the SH3BP2&nbsp;signalsone in&nbsp;innate immunity of nephrotic syndrome.</p>

## Graphical abstract

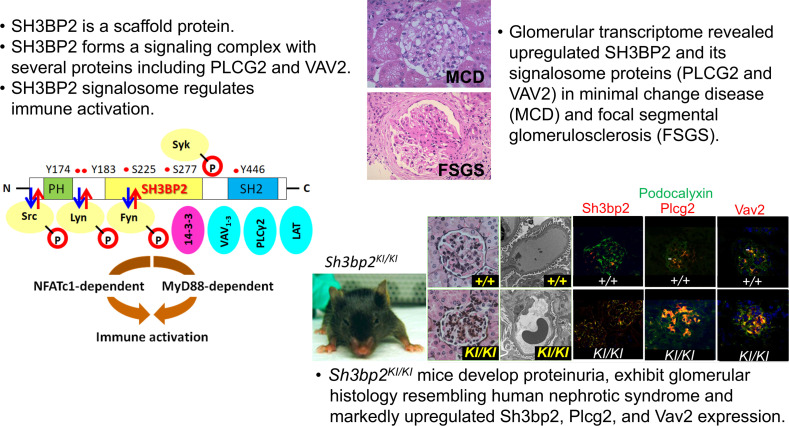

## Introduction

Minimal change disease (MCD) and focal segmental glomerulosclerosis (FSGS) are the 2 most common histological findings and account for approximately 80% of children with idiopathic nephrotic syndrome. Patients with nephrotic syndrome are initially treated with steroids and classified as either steroid sensitive (~85%) or steroid resistant (~15%), and other immunosuppressive drugs are added to the treatment regimen based on the clinical course of the disease (1–3). Overall, immunosuppressants provide considerable disease control in a large proportion of children with nephrotic syndrome (MCD and FSGS). However, the underlying immunopathogenesis remains unknown.

Innate and adaptive immune responses involve distinct mechanisms and interact with each other. Over the years, different aspects of the immune system, including T cells, B cells, and components of the innate immune system, have been implicated in nephrotic syndrome (4–10). Viral RNA/DNA and bacterial cell wall products activate the innate immune system through pattern-recognizing receptors, such as Toll-like receptors (TLRs), resulting in elevated levels of several serum cytokines (11, 12). Viral infections trigger ~70% of relapses and ~45% of self-limiting increase in proteinuria in children with nephrotic syndrome (13). We reported functional TLRs and demonstrated bacterial toxin LPS-induced activation of the TLR4/MyD88/NF-κB signaling pathway in human podocytes (14). LPS upregulates TLR4 expression, disrupts the actin cytoskeleton in mouse podocytes in vitro as well as in vivo, and induces proteinuria and podocyte foot process effacement in SCID mice (devoid of T and B cells), indicating the importance of the innate immune system in nephrotic syndrome (15). A highly complex network of the innate immune system involves cytokines, receptor/nonreceptor proteins, and signaling pathways in both immune and nonimmune cells. Scaffold proteins are nonreceptor proteins that perform a critical role through their ability to bind multiple signaling proteins. Thus, scaffold proteins enable suitable configuration and localization of key molecules that coordinate multiple signaling pathways.

**Figure 1:**
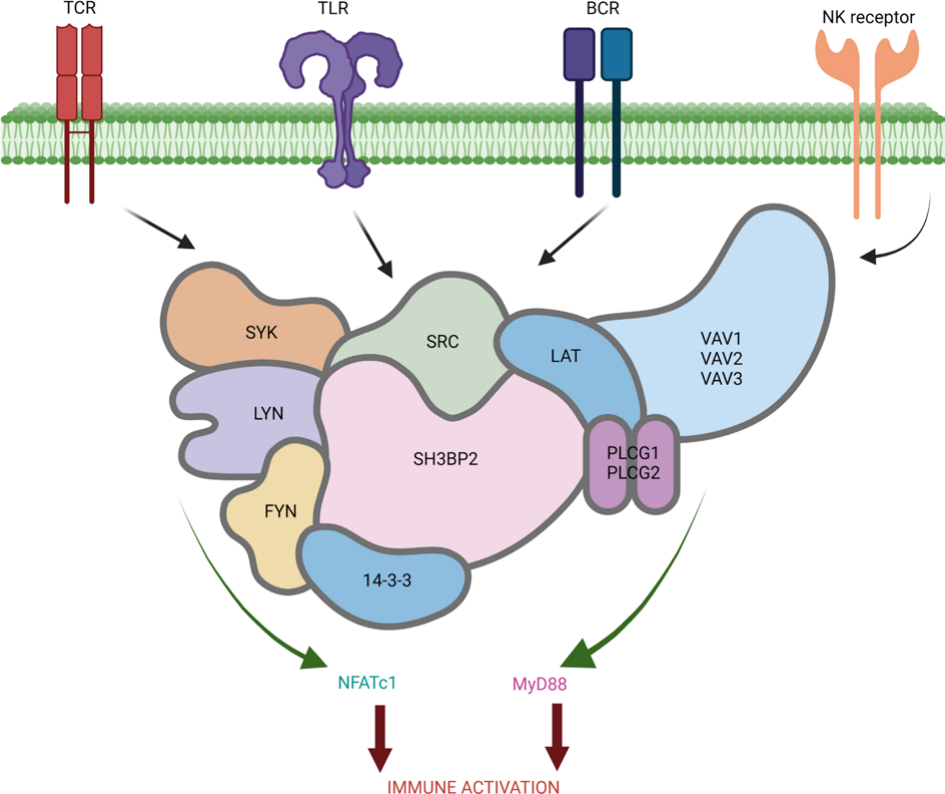
The SH3BP2 “signalosome.” SH3BP2, a cytoplasmic scaffold protein, was originally identified as a protein interacting with the SH3 domain of Abl protein tyrosine kinase. SH3BP2 integrates multiple signaling pathways in T cells, B cells, macrophages, NK cells, etc., by complexing with Src family kinases (LYN and FYN), Syk family kinases, Rho-guanine nucleotide exchange factor VAV (VAV1, VAV2, and VAV3), linker for activation of T cells family member (LAT), phospholipase C gamma (PLCγ1 and PLCγ2), and 14-3-3 group of proteins following activation of T cell receptor (TCR), B cell receptor (BCR), TLRs, or NK receptor. The SH3BP2-mediated downstream activation of nuclear factor of activated T cells 1 (NFATc1) and MyD88 results in immune activation. Created with BioRender.com.

Src homology 3-binding protein 2 (SH3BP2) is a cytoplasmic scaffold protein that forms a signaling complex (signalosome) with SRC, SYK, FYN, phospholipase Cγ (PLCγ), Rho-guanine nucleotide exchange factor VAV1 (VAV1), VAV2, and more to integrate multiple signaling pathways in macrophages and T, B, and NK cells (Figure 1) (16–20). SH3BP2 is required for activation, expansion, and differentiation of T cells involving the calcineurin/NFAT pathway (21–24). SH3BP2 with its binding partners SYK, FYN, PLCγ2, VAV1, and VAV2 (but not BTK) also determine B cell activation mediated by NFAT (25–27). Cytotoxicity of NK cells increase following CD244 activation, resulting in recruitment of SH3BP2 (23, 24). Mice lacking SH3BP2 show suboptimal activation of T and B cells while transgenic mice (*Sh3bp2^KI/KI^*) form hyperactive macrophages and involve the TLR/MyD88 pathway (28–32). We surmised that the SH3BP2 signaling complex, given its role across multiple immune cells, may play an important role in immunopathogenesis of nephrotic syndrome.

We hypothesized that SH3BP2 signaling complex (signalosome) is upregulated in human nephrotic syndrome. We used human transcriptomic and clinical data from the Nephrotic Syndrome Study Network (NEPTUNE) to demonstrate that SH3BP2, its partner molecules (especially PLCγ2 and VAV2), and innate immune signaling pathways are upregulated in the glomerular transcriptome in nephrotic syndrome.

To further validate the role of increased SH3BP2 signaling identified in human data, we identified transgenic mice (*Sh3bp2^KI/KI^*) with a gain-in-function mutation due to a proline-to-arginine substitution in the *Sh3bp2* gene. These animals demonstrate increased innate immune activation and recapitulate human cherubism (30–32). Results show that *Sh3bp2^KI/KI^* mice develop severe albuminuria (~25-fold increase) and altered podocyte ultrastructure with foot process effacement that characterize nephrotic syndrome. *Sh3bp2^KI/KI^* mice also show increased glomerular and podocyte expression of PLCγ2 and VAV2. We further demonstrate that SH3BP2 protein is expressed in human podocytes and co-immunoprecipitates with PLCγ2 and VAV2. SH3BP2 protein is expressed in both immune and nonimmune cells (e.g., glomerular cells) and plays a role in both innate and adaptive immunity. We postulate that SH3BP2 modulates immunopathogenesis of nephrotic syndrome.

## Results

RNA-sequencing (RNA-Seq) data from healthy controls (transplant donors) and participants with MCD or FSGS (NEPTUNE Study) were analyzed using bioinformatic tools. The following outlines the results demonstrating upregulation of SH3BP2, its binding partners, and signaling pathways in individuals with nephrotic syndrome, followed by results using *Sh3bp2^KI/KI^* mice with a gain-in-function mutation demonstrating a phenotype of nephrotic syndrome, and verifying the expression of SH3BP2, PLCγ2, and VAV2 in human and mouse podocytes.

### Bioinformatic studies using human kidney biopsy tissue

#### SH3BP2 signalosome, its downstream molecules, and signaling pathways are upregulated in MCD and FSGS.

**Figure 2:**
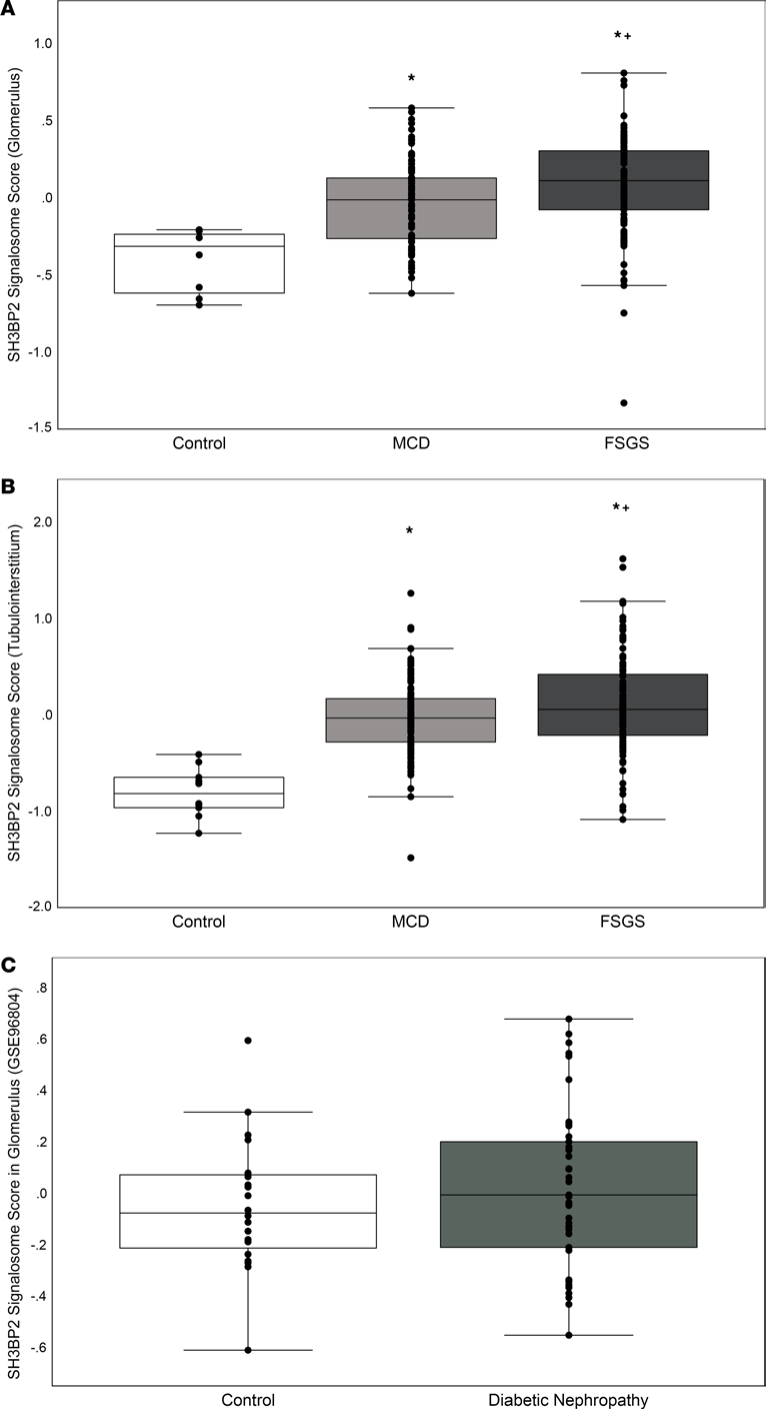
Changes in SH3BP2 signalosome score. Box-and-whisker plots show the distribution of *z* score for the SH3BP2 signaling complex (SH3BP2 signalosome score), which is a composite of 18 genes that form the SH3BP2 signalosome for the mRNA expression in control (*n* = 8 and *n* = 10 for glomerular and tubulointerstitial transcriptome, respectively), MCD (*n* = 89 and *n* = 110 for glomerular and tubulointerstitial transcriptome, respectively), and FSGS (*n* = 93 and *n* = 114 for glomerular and tubulointerstitial transcriptome, respectively) participants. Box plots show the median with the box representing interquartile range (25th to 75th percentile), and 95% of the data are within the limits of the whiskers. Glomerular compartment is shown in **A**, and tubulointerstitial compartment is shown in **B** (see text for details on calculating pathway activation scores). One-way ANOVA followed by Tukey HSD post hoc test was used to compare the mean between the 3 study groups. *P < 0.05 compared with control participants, and ^+^*P* < 0.05 compared with MCD. (**C**) The box plots show the distribution of *z* score in glomerulus for the SH3BP2 signalosome score in diabetic nephropathy (control *n* = 20 and diabetic nephropathy *n* = 41). Data on patients with diabetic nephropathy were obtained from the National Center for Biotechnology Information (NCBI) Gene Expression Omnibus in glomerular transcriptome (GEO Series accession no. GSE96804). The 2-group comparison was performed using 2-tailed Student’s *t* test (*P* = 0.401).

The NEPTUNE data set included sequencing data from the glomerular compartment in 8 controls, 89 patients with MCD, and 93 patients with FSGS. We calculated the SH3BP2 signalosome activation score using the 18 genes for the proteins that are known to form the SH3BP2 signaling complex (Figure 1 and Table 1). A significant increase in the *z* score of the SH3BP2 signalosome in MCD (–0.04 ± 0.27, *P* = 0.001) and in FSGS (0.08 ± 0.33, *P* < 0.001) was observed compared with the control group (–0.41 ± 0.21) (Table 1 and Figure 2). As shown in Table 1, the expression of *SH3BP2*, *VAV1*, *VAV2*, *PLCG2*, *YWHAG*, and *YWHAB* genes was significantly changed in MCD and FSGS. Further, downstream signaling from SH3BP2 involved NFATc1 transcription factor and MyD88 pathways of the innate immune system (Figure 1). Expression of *NFATc1*, *MyD88*, and *TRIF* genes was significantly upregulated in MCD and FSGS and that of *NFATc2* in FSGS only (Table 2). The activation scores for TLR, NLR, cytokine-cytokine receptor, and TNF-α pathways, but not RLR, IL-1β, and measles pathways, were significantly upregulated in FSGS (Table 1).

Tubulointerstitial compartment sequencing data for the SH3BP2 signalosome score, downstream pathways, and genes from 10 controls, 110 patients with MCD, and 114 patients with FSGS in the NEPTUNE data set also showed similar results (Table 2, Table 3, and Figure 2).

Additionally, we compared present results with the effects of diabetic nephropathy on the SH3BP2 signalosome using glomerular transcriptome data (GEO Series accession GSE96804). SH3BP2 signalosome score in the glomerular transcriptome was not significantly changed in proteinuric participants (2.53 ± 1.20 g/d) with diabetic nephropathy (Figure 2).

**Figure 3:**
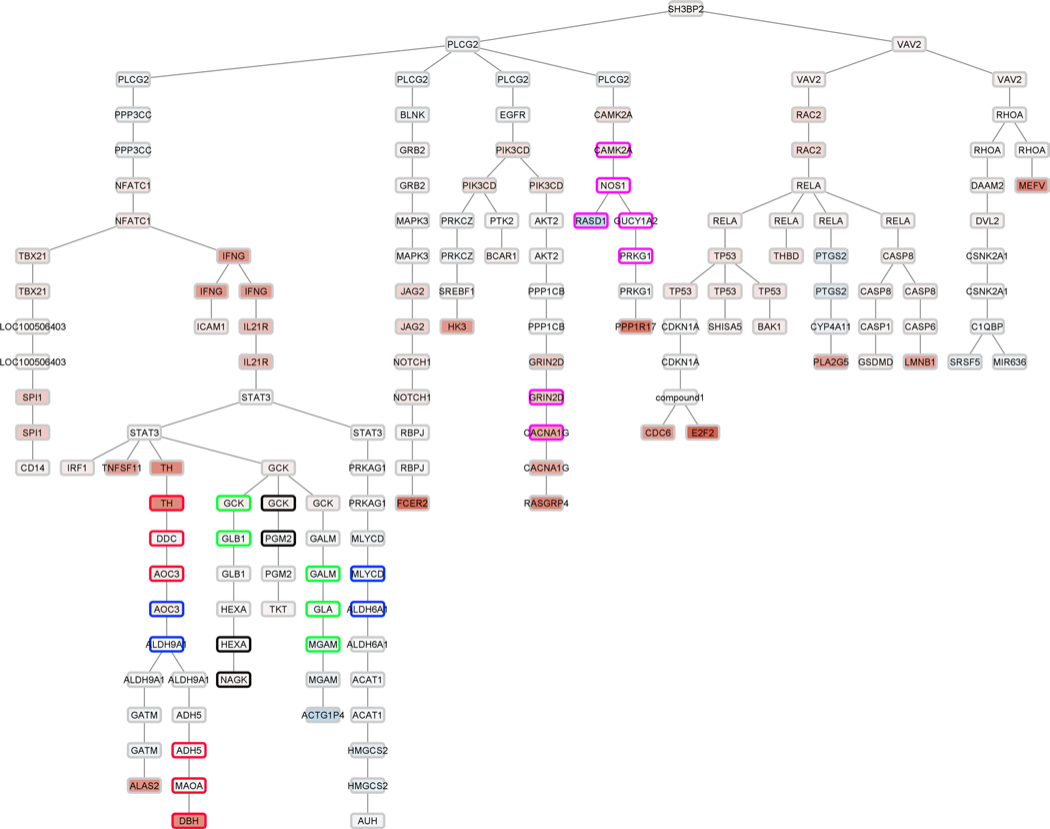
Pathway network map generated using *SH3BP2* as the seed gene in the glomerular compartment of participants in the NEPTUNE data set. The genes belonging to the top 5 significant pathways based on *P* value for the pathways indicated by IMPRes analysis are bordered with the corresponding color. The pathway network analysis shows that SH3BP2-mediated signaling involves PLCγ2 and VAV2.

The NEPTUNE RNA-Seq data were further analyzed using the IMPRes algorithm for in silico visualization of the pathways for *SH3BP2* seed gene in glomerular and tubulointerstitial compartments (Figure 3 and Supplemental Figure 1; supplemental material available online with this article; https://doi.org/10.1172/jci.insight.170055DS1). The IMPRes results revealed that *SH3BP2* engaged *PLCG2* and *VAV2*. *PLCG2* subsequently recruited *NFATC1*, *IFNG*, and *STAT3*, and *VAV2* recruited *RHOA*, *RAC2*, and *RELA* for downstream signaling.

#### Lower SH3BP2 signalosome score is associated with MCD compared with FSGS and with remission and baseline eGFR but not with ESKD or ESKD composite outcome.

The clinical demographic data for the participants in control, MCD, and FSGS groups (Table 4) and for children and adults are shown (Supplemental Table 1). SH3BP2 signalosome score was significantly lower in patients with MCD compared with FSGS, and those in remission, but similar between adults and children (Table 5). Other innate immune pathways were also significantly lower in MCD and in children; some of these pathways were associated with remission in the glomerular transcriptome (Table 5). A significant negative correlation between SH3BP2 signalosome and other innate immune pathway scores and the baseline eGFR, but not with baseline proteinuria, was noted (Table 6). No association between ESKD composite and days to ESKD and SH3BP2 signalosome and other innate immune pathway scores was observed (Supplemental Table 2). The regression tree algorithm analysis performed using these pathway scores for outcomes of ESKD composite and remission is shown in Supplemental Figure 2.

We performed principal component analysis on 8 pathway scores and the glomerular sequencing data set. Principal 1 explained 76.1% of variance, principal 2 explained 9.1%, and both in total explained 85.2%, whereas for the tubular sequencing data set principal 1 explained 89.9% of variance, principal 2 explained 5.5%, and both in total explained 95.4%. We then used principal 1 and principal 2 as 2 predictors for the ESKD composite, and incidence of ESKD and remission, after adjusting for prespecified covariates (i.e., IFTA score, sex, ethnicity, diagnosis, baseline age, eGFR, and UPCR as well as baseline and prior medications renin-angiotensin-aldosterone system inhibitors and immunosuppression). There was no additional improvement in the analyses performed (Supplemental Table 3).

### In vivo studies using Sh3bp2KI/KI mice

#### Urine albumin excretion increases ~6-fold and ~25-fold in Sh3bp2^KI/KI^ mice at 4 and 12 weeks of age, respectively.

**Figure 4:**
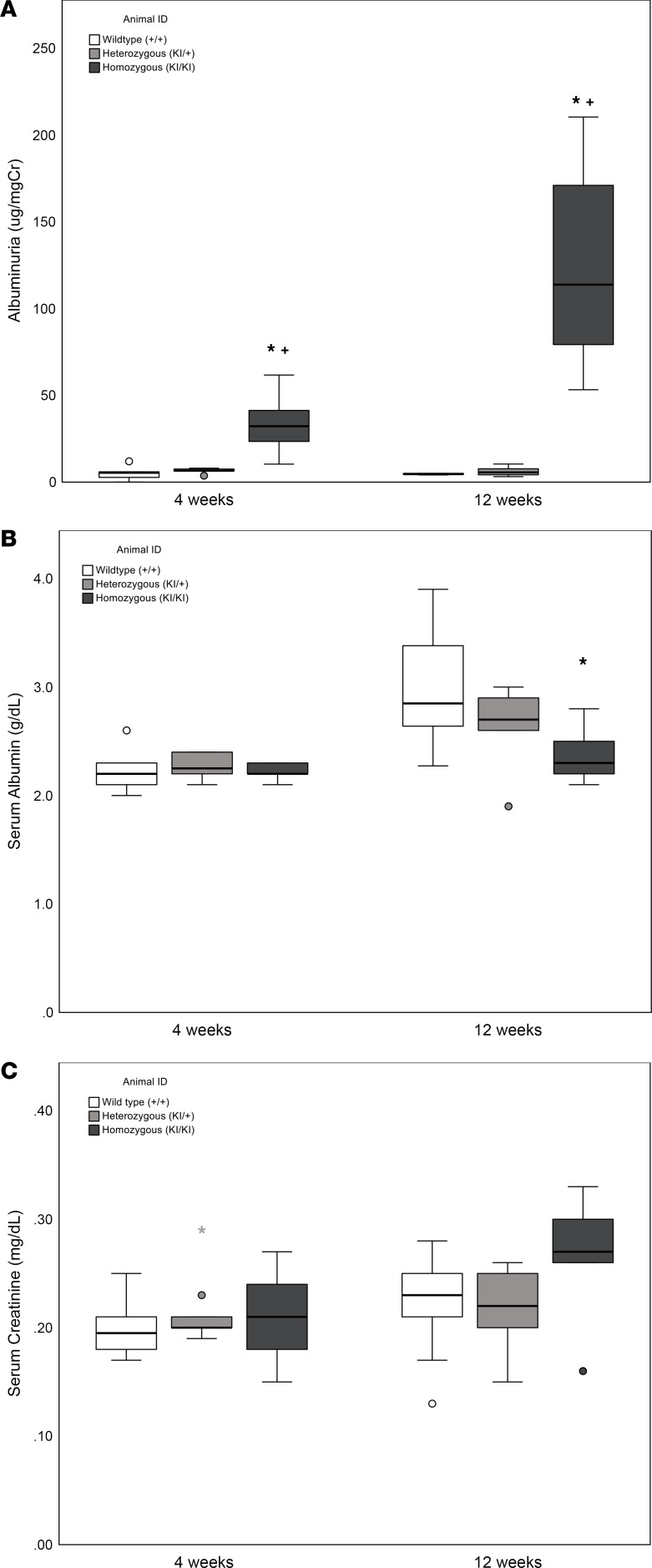
Urine albumin/creatinine ratio, serum albumin, and serum creatinine. Box plots represent (**A**) urine albumin/creatinine ratio (UACR, μg/mgCr), (**B**) serum albumin (g/dL), and (**C**) serum creatinine (mg/dL) in *Sh3bp2* wild-type (*Sh3bp2^+/+^*), heterozygous (*Sh3bp2^KI/+^*), and homozygous (*Sh3bp2^KI/KI^*) mice at 4 and 12 weeks of age. The number of samples is shown in Table 7. Box plots show the median with the box representing interquartile range (25th to 75th percentile), and 95% of the data are within the limits of the whiskers. *P < 0.05 compared with *Sh3bp2^+/+^* animals, and ^+^*P* < 0.05 compared with *Sh3bp2^KI/+^* analyzed by 1-way ANOVA followed by Tukey multiple comparison test.

As shown in Table 7 and Figure 4A, UACR in the wild-type was comparable to heterozygous (*P* = 0.984) but significantly lower than homozygous mice (*P* = 0.005 vs. wild-type and *P* = 0.007 vs. heterozygous) at 4 weeks of age. At 12 weeks, UACR in the wild-type was comparable to heterozygous (*P* = 1.000) and significantly lower than the homozygous (*P* = 0.001 vs. wild-type and *P* = 0.001 vs. heterozygous) mice. Urine creatinine in the wild-type (29.9 ± 12.8 mg/dL) was comparable to heterozygous (34.0 ± 8.7 mg/dL, *P* = 0.849) and homozygous mice (22.5 ± 13.5, *P* = 0.602 vs. wild-type and *P* = 0.312 vs. heterozygous) at 4 weeks of age. At 12 weeks, creatinine in the wild-type (40.0 ± 12.1 mg/dL) was comparable to heterozygous (38.1 ± 19.7 mg/dL, *P* = 0.975) and homozygous mice (26.7 ± 7.5, *P* = 0.328 vs. wild-type and *P* = 0.431 vs. heterozygous).

#### Serum albumin significantly decreases in Sh3bp2^KI/KI^ mice by 12 weeks.

As shown in Table 7 and Figure 4B, serum albumin was not different between the 3 groups of mice at 4 weeks of age. Serum albumin showed a decrease by 12 weeks in heterozygous mice compared with the control group (*P* = 0.124) and was significantly lower in homozygous mice (*P* = 0.002 vs. wild-type and *P* = 0.168 vs. heterozygous).

#### Serum creatinine is unchanged in Sh3bp2^KI/KI^ mice.

Table 7 and Figure 4C show that serum creatinine in *Sh3bp2^KI/KI^* mice at 4 and 12 weeks of age was not significantly different from *Sh3bp2^+/+^* and *Sh3bp2^KI/+^*.

#### Serum cytokines show varying changes in Sh3bp2^KI/KI^ mice.

Table 8 shows serum levels of the 9 cytokines in wild-type, heterozygous, and homozygous mice. Results showed increased TNF-α, IL-6, MCP-1, and IFN-γ at both 4 and 12 weeks and increased IL-2, IL-17, and MIP-1α only at 12 weeks of age. No change was observed in IL-1α and CXCL1 at 4 or 12 weeks of age.

#### Body weight and kidney weight in Sh3bp2 animals.

**Figure 5:**
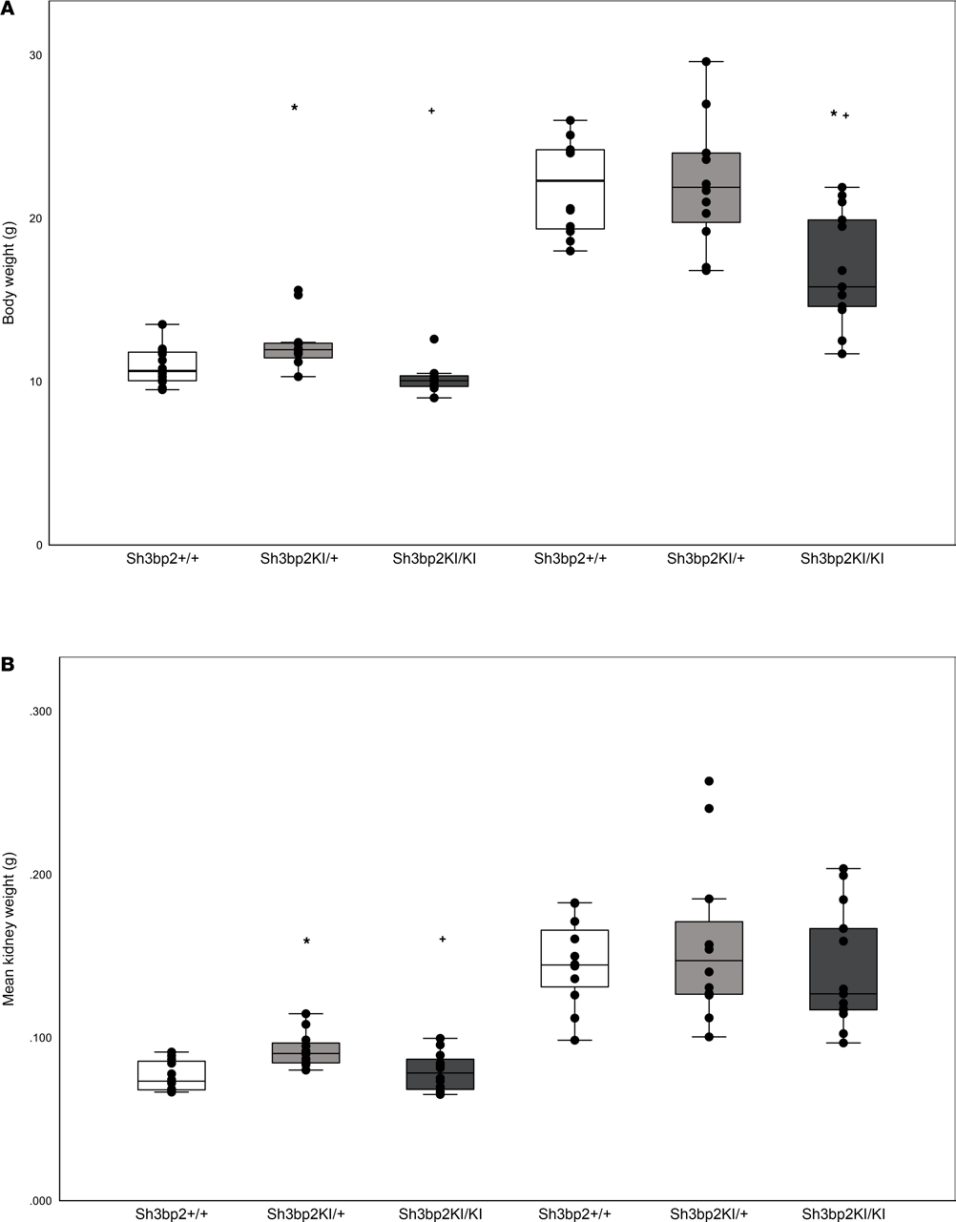
Body weight and kidney weight (average of 2 kidneys/mouse) from wild-type (*Sh3bp2^+/+^*, *n* = 12), heterozygous (*Sh3bp2^KI/+^*, *n* = 12), and homozygous (*Sh3bp2^KI/KI^*, *n* = 12) mice at 4 weeks and 12 weeks of age. Box-and-whisker plots represent body weight (g) (**A**) and kidney weight (g) (**B**) in *Sh3bp2* wild-type (*Sh3bp2^+/+^*), heterozygous (*Sh3bp2^KI/+^*), and homozygous (*Sh3bp2^KI/KI^*) mice at 4 and 12 weeks of age. Box plots show the median with the box representing interquartile range (25th to 75th percentile), and 95% of the data are within the limits of the whiskers. *P < 0.05 compared with *Sh3bp2^+/+^* animals, and ^+^*P* < 0.05 compared with *Sh3bp2^KI/+^* analyzed by 1-way ANOVA followed by Tukey multiple comparison test.

The body weight and kidney weight from wild-type, heterozygous, and homozygous mice at 4 weeks and 12 weeks of age are shown in Table 9 and Figure 5. The body weight of *Sh3bp2^KI/KI^* mice and their kidney weight were slightly lower than those of *Sh3bp2^+/+^* mice, except for a significant decrease in body weight at 12 weeks of age in *Sh3bp2^KI/KI^* mice. *Sh3bp2^KI/+^* mice exhibited the highest body weight and kidney weight.

#### Altered renal morphology and podocyte ultrastructure in Sh3bp2^KI/KI^ mice.

**Figure 6:**
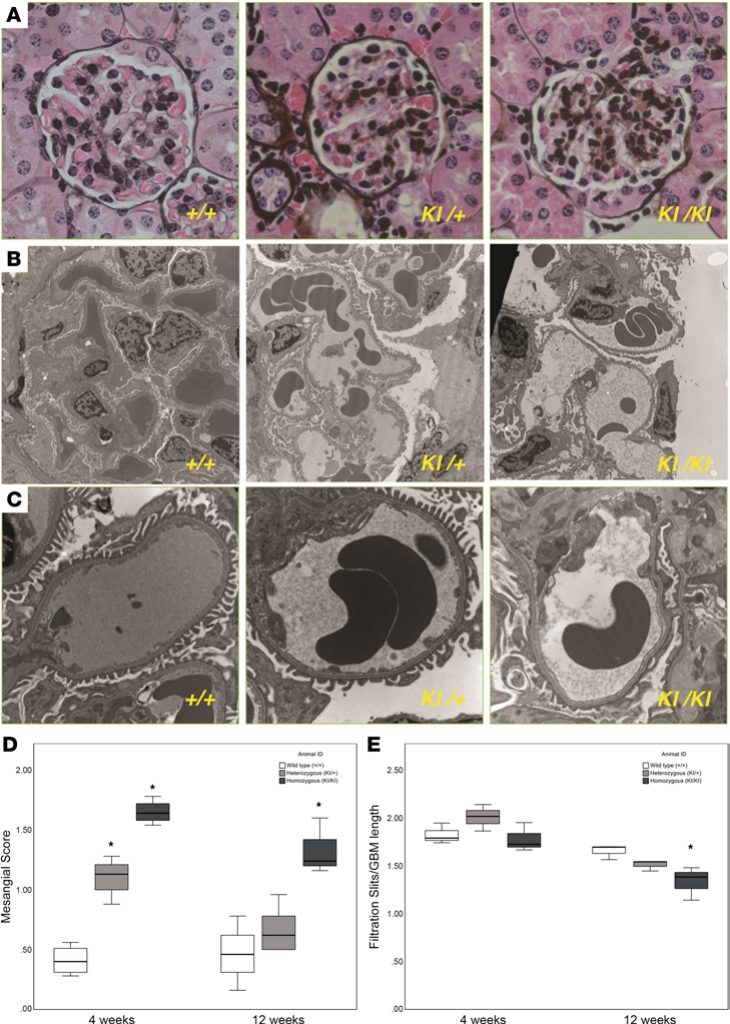
Light and electron microscopy results showing glomerular changes in kidneys from *Sh3bp2^KI/KI^* mice in wild-type (*Sh3bp2^+/+^*), heterozygous (*Sh3bp2^KI/+^*), and homozygous (*Sh3bp2^KI/KI^*) mice. (**A**) Light microscopy showing increased mesangial cellularity in the *Sh3bp2^KI/KI^* mice on silver stain obtained at 400× original magnification at 12 weeks of age. (**B**) Electron microscopy (original magnification, 5,000×) showing absence of electron-dense deposits in the mesangial areas. Higher resolution (original magnification, 15,000×) imaging shows foot process effacement and loss of slit diaphragm in the podocytes in the *Sh3bp2^KI/KI^* mice (**C**). (**D** and **E**) Box plots represent the mesangial score (light microscopy) and the number of filtration slits per glomerular basement length (nm, electron microscopy). Box plots show the median with the box representing interquartile range (25th to 75th percentile), and 95% of the data are within the limits of the whiskers. The number of samples is shown in Table 10. *P < 0.05 compared with *Sh3bp2^+/+^* animals analyzed by 1-way ANOVA followed by Tukey multiple comparison test.

Light microscopy showed normal kidney morphology except for mesangial hypercellularity in the *Sh3bp2^KI/KI^* mice. Semiquantitative analysis revealed increased mesangial score in the *Sh3bp2^KI/KI^* group compared with the wild-type at 4 and 12 weeks, with more marked changes at 4 weeks (Table 10). Figure 6 shows changes in mesangial score in transgenic mice at 4 and 12 weeks of age. The tubulointerstitial and vascular compartments appeared normal.

Electron microscopy showed comparable ultrastructure in the wild-type and the heterozygous *Sh3bp2* mice. However, *Sh3bp2^KI/KI^* mice showed increased mesangial matrix and patchy fusion of foot processes without electron-dense deposits (Figure 6). The number of filtration slits per glomerular basement membrane was significantly decreased by 12 weeks in *Sh3bp2^KI/KI^* mice compared with wild-type (Table 10).

#### Sh3bp2^KI/KI^ mice show increased glomerular expression of PLCγ2 and VAV2.

**Figure 7:**
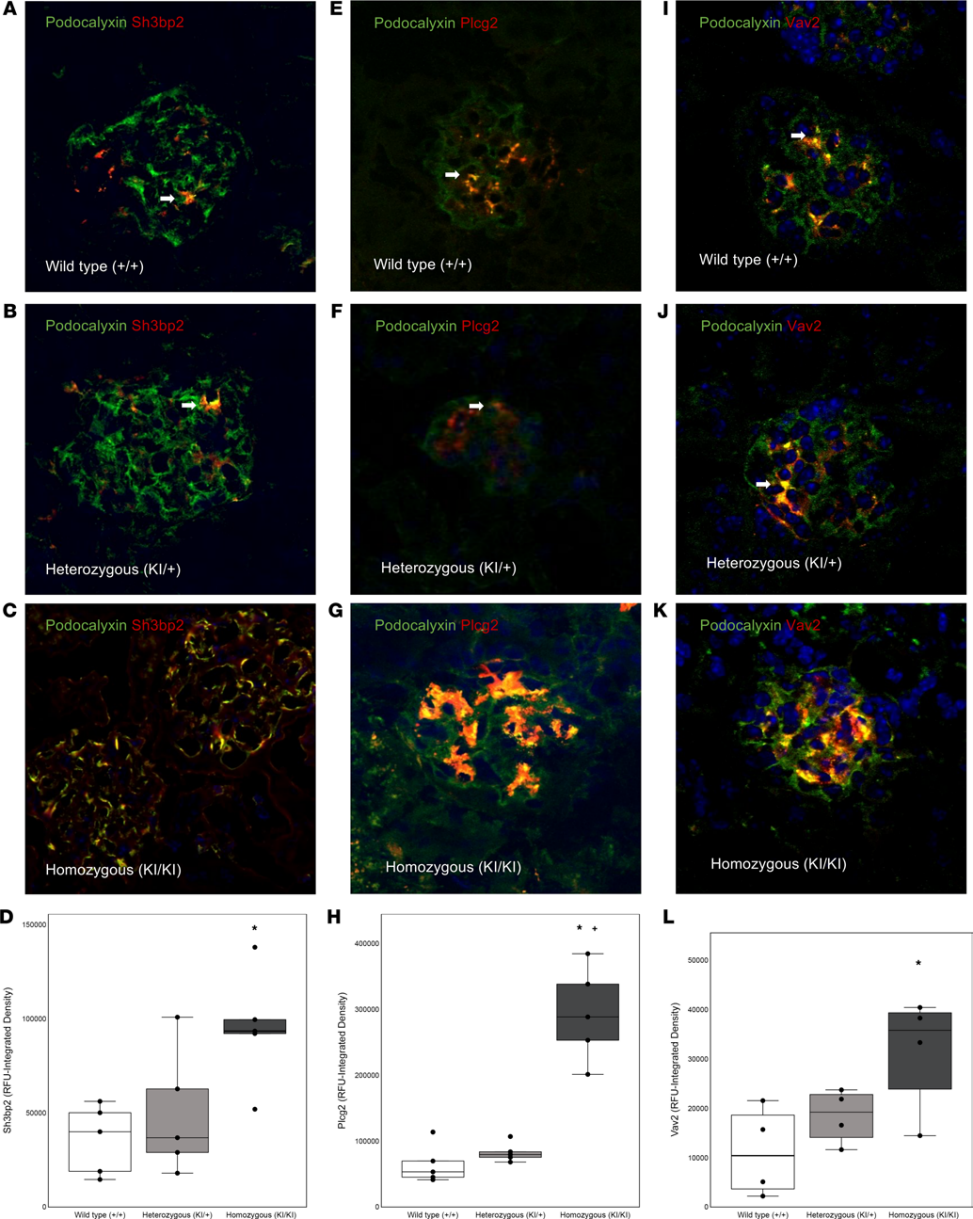
Immunofluorescence microscopy of glomeruli from *Sh3bp2^KI/KI^* mice. Immunofluorescence shows increased SH3BP2 (left, **A**–**D**), PLCγ2 (middle, **E**–**H**), and VAV2 (right, **I**–**L**) expression in the glomeruli from 12-week-old *Sh3bp2^KI/KI^* mice (*n* = 5) compared with control wild-type (*Sh3bp2^+/+^*) mice (*n* = 5) and heterozygous (*Sh3bp2^KI/+^*) mice (*n* = 5). Podocytes were stained for podocalyxin (green fluorescence) while SH3BP2, PLCγ2, and VAV2 were stained with red fluorescence. The red staining demonstrates presence of SH3BP2, PLCγ2, and VAV2 in mesangial cells while orange/yellow staining represents staining within the podocyte. White arrows mark the expression of SH3BP2, PLCγ2, and VAV2 in podocytes in wild-type (*Sh3bp2^+/+^*) and heterozygous (*Sh3bp2^KI/+^*) mice. All confocal images were taken at fixed acquisition settings. Original images were obtained at 630× total magnification. Box plots (**D**, **H**, and **L**) show fluorescence intensity analysis with the median and the box representing interquartile range (25th to 75th percentile), and 95% of the data were within the limits of the whiskers. One-way ANOVA followed by Tukey HSD post hoc test were used to compare the mean values in the 3 groups (*n* = 5/group each). *P < 0.05 compared with *Sh3bp2^+/+^* animals, and ^+^*P* < 0.05 compared with *Sh3bp2^KI/+^* analyzed by 1-way ANOVA followed by Tukey multiple comparison test.

The IMPRes analysis (Figure 3) predicted that *SH3BP2* engages *PLCG2* and *VAV2*. The *Sh3bp2^KI/KI^* mice at 12 weeks of age showed increased expression of PLCγ2 and VAV2 in glomeruli and podocytes (Figure 7 and Table 11), suggesting that SH3BP2 engages PLCγ2 for downstream recruitment of NFATc1, IFN-γ, and STAT3 and engages VAV2 for balance between RHO and RAC.

### In vitro studies using human podocytes

#### Human podocytes demonstrate expression and binding of SH3BP2 to PLCγ2 and VAV2.

**Figure 8:**
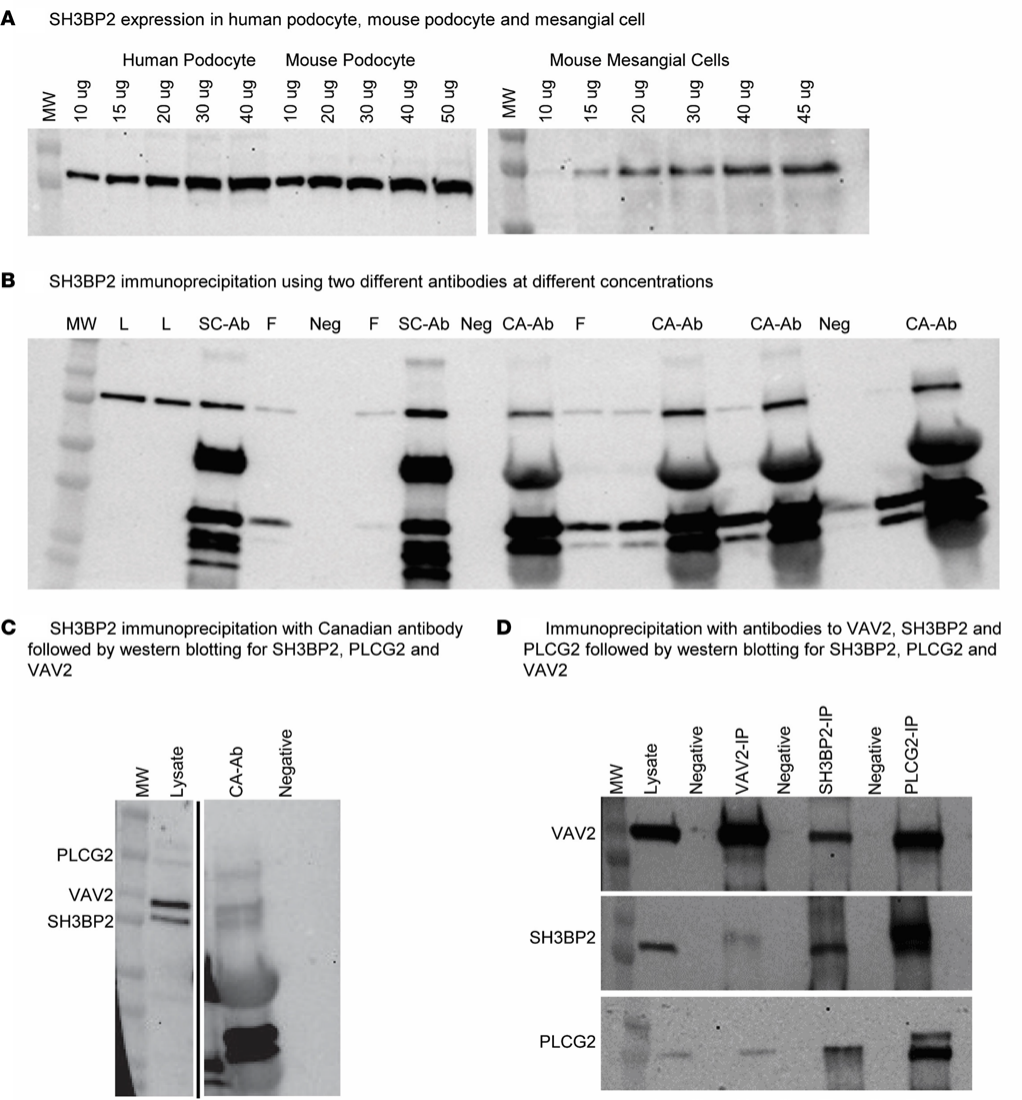
Western blot analysis using total protein lysate from human podocytes. (**A**) Western blotting of protein lysate from human podocytes, mouse podocytes, and mouse mesangial cells shows expression of SH3BP2 protein in podocytes and mesangial cells. (**B**) Western blotting for SH3BP2 was performed with antibody for SH3BP2 (sc-377020, Santa Cruz Biotechnology, and antibody provided as a gift from University of Toronto, Toronto, Ontario, Canada) in different concentrations to optimize the immunoprecipitation experiments. Western blotting showed presence of SH3BP2 in human podocyte cell lysate and its immunoprecipitation with both antibodies. (**C**) The Western blot showing presence of SH3BP2, PLCγ2, and VAV2 in human podocytes. Anti-SH3BP2 antibody (from University of Toronto, Toronto, Ontario, Canada) pulled down VAV2 and PLCγ2. The lanes were run on the same gel but were noncontiguous (separated by black line). (**D**) Immunoprecipitation with VAV2 (sc-271442) antibody, SH3BP2 antibody (sc-377020), and PLCγ2 (MAB3716) antibody followed by Western blotting shows co-precipitation of VAV2, SH3BP2, and PLCγ2. Immunoprecipitation using antibodies against each protein (lane labels on top) followed by Western blotting to probe each protein (indicated on left edge of gel images **C** and **D**) showed that SH3BP2-VAV2-PLCγ2 bound one another in untreated human podocytes. L, protein cell lysate; SC-Ab/SC, Santa Cruz Biotechnology (sc-377020) antibody; CA-Ab/CA, Canadian antibody against SH3BP2 from University of Toronto, Toronto, Ontario, Canada; F, flow through following immunoprecipitation; Neg, negative control; SH3BP2, Src homology 3-binding protein 2; VAV2, Rho-guanine nucleotide exchange factor VAV2; PLCγ2, phospholipase Cγ2; VAV2-IP, immunoprecipitation with VAV2 antibody; SH3BP2-IP, immunoprecipitation with SH3BP2 antibody; PLCγ2-IP, immunoprecipitation with PLCγ2 antibody.

Western blotting of protein lysate from human podocytes showed expression of SH3BP2 (Figure 8A). In addition, we verified the expression of SH3BP2 in mouse podocytes and mouse mesangial cells (Figure 8A). We used 2 antibodies against SH3BP2 to optimize the immunoprecipitation experiments (Figure 8B). Immunoprecipitation with SH3BP2 antibody pulled down both PLCγ2 and VAV2 with it (Figure 8, C and D). In separate experiments, this was further verified by immunoprecipitation using antibodies against VAV2 antibody and PLCγ2 followed by Western blotting. These experiments verified that VAV2, PLCγ2, and SH3BP2 immunoprecipitated together (Figure 8D). Thus, SH3BP2-VAV2-PLCγ2 are bound to each other in unstimulated normal human podocytes.

## Discussion

Clinical efficacy of immunosuppressive agents to control proteinuria in adults and children with MCD and FSGS and other evidence suggest innate immune activation in nephrotic syndrome. Corroborating evidence includes (i) frequent relapses due to viral or bacterial infection, (ii) LPS-induced proteinuria and podocyte foot process effacement in mice lacking T and B cells, (iii) expression of TLRs and the TLR4/MyD88/NF-κB signaling pathway in human podocytes, and (iv) absence of inflammatory cells, complement proteins, or immune deposits (except occasional IgM) in MCD and FSGS (13–15, 33–36). Additionally, communication with the adaptive immune system as well as the presence of receptor/nonreceptor proteins in immune and nonimmune cells constitute a complex network of the immune system. Present results introduce SH3BP2 as a noncatalytic, nonreceptor scaffold protein that integrates multiple immune signaling pathways in immune and nonimmune cells. SH3BP2 signalosome molecules and downstream signaling pathways are upregulated in the glomerular transcriptome in patients with MCD and FSGS (Table 1). Transgenic mice (*Sh3bp2^KI/KI^*) with a gain-in-function mutation and an upregulated innate immune system demonstrated laboratory and histological features of nephrotic syndrome. In vitro studies validated the expression of SH3BP2 and its binding partners in human and mouse podocytes. These results highlight the importance of SH3BP2 in nephrotic syndrome.

The role of SH3BP2 in glomerular or podocyte function is not known but has been demonstrated in the activation of macrophages and T, B, and NK cells (19, 20). MHC/peptide complex binding to TCR is associated with SH3BP2 phosphorylation, resulting in activation, expansion, and differentiation via the calcineurin/NFAT and Ras-dependent pathways (21–24). SH3BP2 with its binding partners SYK, FYN, PLCγ2, VAV1, and VAV2 is required for B cell activation mediated by NFAT (25–27). Mice lacking SH3BP2 show suboptimal activation of T and B cells (28, 29). Myeloid progenitor cells from *Sh3bp2^KI/KI^* mice (i) yield hyperactive macrophages mediated by ERK in the presence of M-CSF and (ii) generate osteoclasts mediated by Syk in the presence of RANKL (30–32). NK cell receptor 2B4 activation followed by recruitment of SH3BP2 and VAV1 results in increased NK cell cytotoxicity (37, 38).

The ability of SH3BP2 to influence multiple signaling events is evident as it forms a signalosome complex involving several proteins (Figure 1). Calculated activation scores using previously described methods revealed significantly elevated SH3BP2 signalosome score and the upregulated signalosome genes in MCD and FSGS (Table 1) (39–41). Significantly increased pathway activation scores for TLR and NLR pathways, but not RLR, were also identified. MyD88 and TRIF gene expression downstream of TLR and NLR was also increased in MCD and FSGS (Table 2). We have previously reported expression of TLRs (TLR4/MyD88/NF-κB pathway) in human podocytes (14). TLR4 ligand LPS was found to disrupt the actin cytoskeleton, increase free radical generation, and activate NF-κB in vitro (15, 42). LPS administration to mice resulted in foot process effacement and albuminuria (15, 43, 44). Similarly, Poly(I:C), a TLR3 ligand, also caused significant proteinuria in mice (45). Thus, SH3BP2 upregulation is closely associated with innate immune signaling.

The cytokine-cytokine receptor pathway score indicates upregulation of cytokines, their receptors, and signaling pathways (Table 1). It corroborates the importance of cytokines in nephrotic syndrome and provides insights into other pathways. For example, measles infection is associated with virus-induced suppression of the innate immune system (46, 47); it also induces remission in children with nephrotic syndrome with overactive innate immune system (48–50). Therefore, unchanged measles pathway score in nephrotic syndrome indirectly supports the role of innate immune system in nephrotic syndrome. Thus, upregulated SH3BP2, TNF-α, TLR, NLR, and cytokine-cytokine receptor pathway scores and unchanged scores for measles, RLR, and IL-1β pathways in human nephrotic syndrome indicate an important role of SH3BP2-mediated innate immune activation. Present results are focused on glomerular transcriptome, but the role of the tubular compartment is important and has been comprehensively discussed in recent reviews (51, 52). SH3BP2 is expressed in both immune and nonimmune cells, and its upregulation in the tubulointerstitial transcriptome would be anticipated in nephrotic syndrome (Supplemental Table 3). Results of tubulointerstitial transcriptome analysis paralleled those of the glomerular transcriptome (Table 3 and Figure 2).

Increased pathway activation scores provided the rationale for a stepwise in silico analysis of signaling pathways downstream of *SH3BP2* (seed gene) using the IMPRes algorithm. Previously, we used this tool to identify pathways involved in hyperfiltration-mediated glomerular injury (53). Human glomerular transcriptome data (Figure 3) suggest that *SH3BP2* recruits *PLCG2* and *VAV2* in downstream signaling in nephrotic syndrome. We then verified (*vide infra*) this relationship between SH3BP2, PLCγ2, and VAV2 in *Sh2bp2^KI/KI^* mice (Figure 7) and in human podocytes in vitro (Figure 8).

SH3BP2-mediated immune activation is likely more important in disease activity than in disease progression (Tables 5 and 6) since lower SH3BP2 signalosome score was associated with remission, MCD, and higher eGFR but not with ESKD. Additionally, increased SH3BP2 signaling does not appear to be a generalized change across common proteinuric disease conditions as glomerular transcriptome data in diabetic nephropathy (GEO GSE96804) showed unchanged SH3BP2 signalosome score (Figure 2). To explore the importance of SH3BP2-mediated signaling in nephrotic syndrome, we identified a gain-in-function transgenic *Sh3bp2^KI/KI^* mouse that was previously used to study the innate immune system in human cherubism.

*Sh3bp2^KI/KI^* mice demonstrated high albuminuria and hypoalbuminemia with unchanged serum creatinine by 12 weeks of age (Figure 4). Additionally, renal histology of the *Sh3bp2^KI/KI^* mice showed increased mesangial cellularity, and electron microscopy revealed foot process effacement with a significant decrease in the number of slit diaphragms without electron-dense deposits (Figure 6). Mild focal mesangial prominence (≤ 3–4 cells/segment) in MCD and > 4 mesangial cells/segment in MCD variant (diffuse mesangial hypercellularity) have been described (54). *Sh3bp2^KI/KI^* mice also showed an altered serum cytokine profile by 12 weeks of age indicating immune dysregulation (Table 8). Nephrotic syndrome is consistently associated with elevated levels of serum IL-2, IL-2R, IFN-γ, and TNF-α and normal levels of IL-1α, IL-1β, and IFN-α (11, 12, 55–57). In a few studies, serum IL-6 and IL-17 were found to be elevated while MCP-1 was normal in nephrotic syndrome (58, 59). Thus, all common histological and laboratory findings that characterize human nephrotic syndrome were observed in *Sh3bp2^KI/KI^* mice.

In addition to the nephrotic syndrome phenotype identified in this study, *Sh3bp2^KI/KI^* mice were reported to exhibit a cherubism phenotype. Although these are distinct clinical entities, their co-occurrence in *Sh3bp2^KI/KI^* mice presents an interesting finding. The clinical course of cherubism is similar to nephrotic syndrome in children. Facial swelling due to cysts in the mandible and maxilla in children with cherubism manifests at about 3 years of age and starts to regress after puberty (30). Similarly, nephrotic syndrome in children peaks between 2 and 6 years, and relapse of nephrotic syndrome becomes infrequent with age (54). The cherubism phenotype in *Sh3bp2^KI/KI^* mice was rescued when crossed with TNF-α^−/−^ or MyD88^−/−^, but not with Rag1^–/–^, mice (lacking T and B cells) (31). Further, crossing *Sh3bp2^KI/KI^* mice with TLR2^−/−^, TLR4^−/−^, and TLR2^−/−^ TLR4^−/−^ mice, but not with IL-1R^−/−^ mice, rescued the cherubism phenotype (32). Increased TNF-α pathway score and unchanged IL-1β score in patients with FSGS corresponded with results of crossing *Sh3bp2^KI/KI^* mice with TNF-α^−/−^ and IL-1R^−/−^ mice, respectively (Table 1). Thus, immune activation in *Sh3bp2^KI/KI^* is driven predominantly by innate immune system independent of T and B cells (30–32).

Immunofluorescence microscopy of *Sh2bp2^KI/KI^* mouse kidneys validated the importance of PLCγ2 and VAV2 downstream of SH3BP2 in nephrotic syndrome as suggested by in silico IMPRes analysis of human glomerular transcriptome (Figure 7 and Table 11). Expression of and interaction between these proteins were observed in human and mouse podocytes (Figure 8). PLCγ2 hydrolyzes the membrane phospholipid phosphatidylinositol 4,5-bisphosphate to the secondary messengers inositol 1,4,5-trisphosphate and diacylglycerol (60). PLCγ2 missense coding variants have been identified as candidate loci in genome-wide association study of children with steroid-sensitive nephrotic syndrome (61). VAV2 (DBL family of proteins) is a ubiquitous guanine nucleotide exchange factor for the small GTPase RAC1 and extensively studied for its role in neurite outgrowth and branching and tumor cell invasion (62–65). Balance between Rac and Rho GTPases determines Src-mediated tyrosine phosphorylation and degradation of synaptopodin; Vav2 activation was found to cause enhanced Rac1 signaling and ultimate loss of stress fibers (66, 67). Thus, SH3BP2-VAV2 maybe involved in the immune-mediated podocyte actin cytoskeleton disruption in nephrotic syndrome. Also, recruitment of PLCγ2 by SH3BP2 results in activation of NFATc1, IFN-γ, and STAT3, among others. NFATc1 is the target for calcineurin inhibitors used for treating nephrotic syndrome, serum IFN-γ is increased in nephrotic syndrome, and JAK/STAT signaling is important in FSGS and in recurrence of FSGS following transplantation (11, 12, 68, 69).

These findings provide promising possibilities to study a multifaceted role of scaffold protein SH3BP2 in podocyte injury and disruption of the glomerular filtration barrier in nephrotic syndrome. We postulate 3 possibilities: (a) the direct involvement of SH3BP2 activation in podocytes, (b) a paracrine interaction between mesangial cells and podocytes, or (c) the influence of pro-inflammatory cytokines emanating from circulating immune cells. First, increased expression scores for SH3BP2, its binding partners (PLCγ2, VAV2, etc.), and related signaling pathways in MCD and FSGS (glomerular transcriptome data) in humans was corroborated by upregulated *Sh3bp2*, *Plcg2*, and *Vav2* in *Sh3bp2^KI/KI^* mice as well in in vitro studies using human podocytes. Upregulation of PLCγ2 and VAV2 with SH3BP2 is noteworthy as PLCγ2 is known to mediate the downstream activation of NFATc1 and STAT3 signaling in podocytes while VAV2 mediates the RhoA/Rac1 imbalance resulting in actin cytoskeleton disruption, supporting a role for direct role for podocytes (66, 67). Second, mild mesangial hypercellularity in *Sh3bp2^KI/KI^* mice corresponds with increased mesangial cellularity in patients with nephrotic syndrome. Upregulated SH3BP2 and its signalosome constituents in mesangial cells may result in paracrine interaction with podocytes, resulting in injury. Further, circulating pro-inflammatory cytokines from immune cells may be a third cause of injury to podocytes. Serum cytokines are increased in children with nephrotic syndrome as well as in *Sh3bp2^KI/KI^* mice (11, 12, 55–59, 70). Podocytes express receptors for TNF-α and IL-6, and we reported increased glomerular albumin permeability by TNF-α and IL-6 (71, 72). Thus, present studies provide evidence for scaffold protein SH3BP2 in immunopathogenesis of nephrotic syndrome, and a detailed investigation is needed in future studies to delineate the functional involvement of SH3BP2 and its binding partners in immune activation altering the podocyte ultrastructure and glomerular function. Thus *Sh3bp2^KI/KI^* complemented with *Sh3bp2^–/–^* mice for in vivo studies and human podocytes for in vitro studies will be valuable to investigate immune dysregulation in nephrotic syndrome.

## Methods

The Supplemental Methods section provides details of methods outlined below.

### Analysis of transcriptomic data (NEPTUNE Study).

Transcriptomic analysis of human kidney tissues was performed at the University of Michigan Advanced Genomics Core (Ann Arbor, Michigan, USA) as previously described (39–41). RNA-Seq data from glomerular and tubulointerstitial compartments from biopsy-proven MCD or FSGS participants in the NEPTUNE Study (ClinicalTrials.gov NCT1209000) were used (73). NEPTUNE demographic and clinical variables were used for outcome analysis (Supplemental Methods, A and B). The gene expression values were *z*-transformed, and *z* scores were calculated as previously described for JAK/STAT and TNF-α pathway scores (74, 75). The Kyoto Encyclopedia of Genes and Genomes database was curated for pathways known to be associated with innate immunity. *Sh3bp2*-linked genes and signaling pathways were curated from literature for SH3BP2 signalosome, IL-1β (inflammasome), and TNF-α scores (40, 41). A list of genes associated with IL-1β and TNF-α scores curated from literature is provided in Supplemental Tables 4 and 5. The IMPRes algorithm was used for stepwise pathway exploration starting with *SH3BP2* as seed gene (76, 77) (Supplemental Methods C). To further evaluate the relevance of SH3BP2 signalosome score in another noninflammatory proteinuric kidney disease, we searched for available glomerular transcriptomic data on diabetic nephropathy available in NCBI GEO.

### Sh3bp2-transgenic mice.

*Sh3bp2^KI/KI^* mice carry a proline-to-arginine (P416R) substitution in exon 9 of murine *Sh3bp2* gene on the C57BL6/J background. Heterozygous *Sh3bp2^KI/+^* mice (University of Missouri, Kansas City, Missouri, USA) were used for breeding to obtain *Sh3bp2^KI/KI^* animals (ref 30; Supplemental Methods D). Kidneys, blood, and urine from 4-week-old and 12-week-old *Sh3bp2^KI/KI^* (homozygous), *Sh3bp2^KI/+^* (heterozygous), and *Sh3bp2^+/+^* (wild-type) mice were harvested for analysis. Urine albumin and creatinine and serum albumin were measured using commercially available kits (Supplemental Methods E). Levels of specific serum cytokines (TNF-α, IL-6, MCP-1, IL-2, IFN-γ, IL-17, MIP-1α, IL-1α, and CXCL1) were assayed using a Luminex 200 platform (Invitrogen). (Supplemental Methods F). Kidney histology was assessed using paraffin-embedded tissue sections to determine changes in glomerular characteristics (mesangial matrix and mesangial cellularity). Glutaraldehyde-fixed tissue samples were used to assess changes in podocyte morphology by electron microscopy. Expression for podocalyxin, PLCγ2, and VAV2 was evaluated in kidney tissue using immunofluorescence microscopy (Supplemental Methods G).

### Cell culture, immunoprecipitation, and Western blotting.

Differentiated conditionally immortalized human and mouse podocytes and mesangial cells were used. Protein expression for SH3BP2, PLCγ2, and VAV2 were evaluated using co-immunoprecipitation and Western blotting. Details are provided under Supplemental Methods H and I.

### Statistics.

Details of statistical tools used for experiments using animals and for clinical outcome in the NEPTUNE participants are provided under Results and in Supplemental Methods J. One-way ANOVA followed by Tukey HSD post hoc test was used to compare the mean between the 3 study groups, and 2-tailed Student’s *t* test was used for 2-group comparisons using the SPSS 24 statistical software (IBM). A *P* value less than 0.05 was considered significant.

### Study approval.

Protocols for using mice were approved by the Institutional Animal Care and Use Committee at the Kansas City VA Medical Center, Kansas City, Missouri, USA (Mouse Model of Kidney Disease-1, ID: 00680, Protocol: MS0003-A). Animals were maintained at Association for Assessment and Accreditation of Laboratory Animal Care International–approved facilities with unrestricted access to food and water under a 12-hour light/12-hour dark cycle. The study was carried out in compliance with the Animal Research: Reporting of In Vivo Experiments guidelines. With respect to the human data, consent was obtained prior to participating in the NEPTUNE Study (ClinicalTrials.gov NCT1209000), and the data were made available by the Data Analysis and Coordinating Center (DACC) in a deidentified manner for analysis.

### Data availability.

The human data used in this study are from the NEPTUNE. NEPTUNE data are available upon request and approval of an ancillary study application from the DACC (https://www.neptune-study.org/). The animal data are available in the Supporting Data Values file.

## Author contributions

TS, RS, LHM, JBH, CS, RR, and MS designed the study; JZ, VCB, MHR, SS, and DE carried out experiments; TS, REG, TJ, YJ, VSS, DSG, WH, CS, YW, LHM, JBH, RR, and MS analyzed the data; SS performed cell culture experiments; TY and YU developed mice and collected samples for the study; TS, TJ, YJ, and MS made the figures; TS and MS drafted and revised the paper; and all authors approved the final version of the manuscript.

## Supplementary Material

Supplemental data

Supporting data values

## Figures and Tables

**Table 11:**
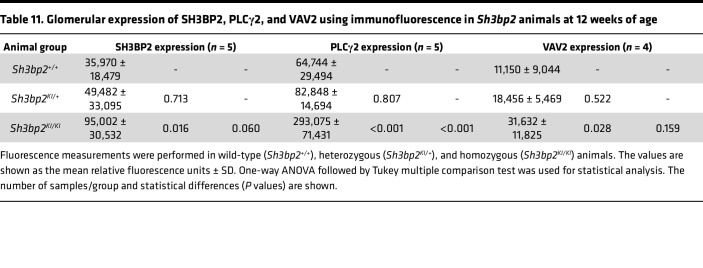
Glomerular expression of SH3BP2, PLCγ2, and VAV2 using immunofluorescence in *Sh3bp2* animals at 12 weeks of age

**Table 10:**
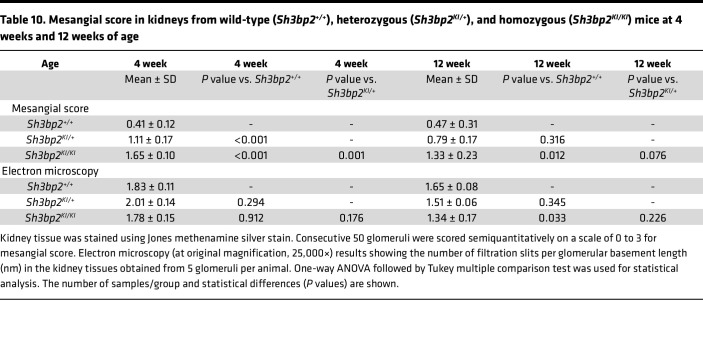
Mesangial score in kidneys from wild-type (*Sh3bp2^+/+^*), heterozygous (*Sh3bp2^KI/+^*), and homozygous (*Sh3bp2^KI/KI^*) mice at 4 weeks and 12 weeks of age

**Table 4:**
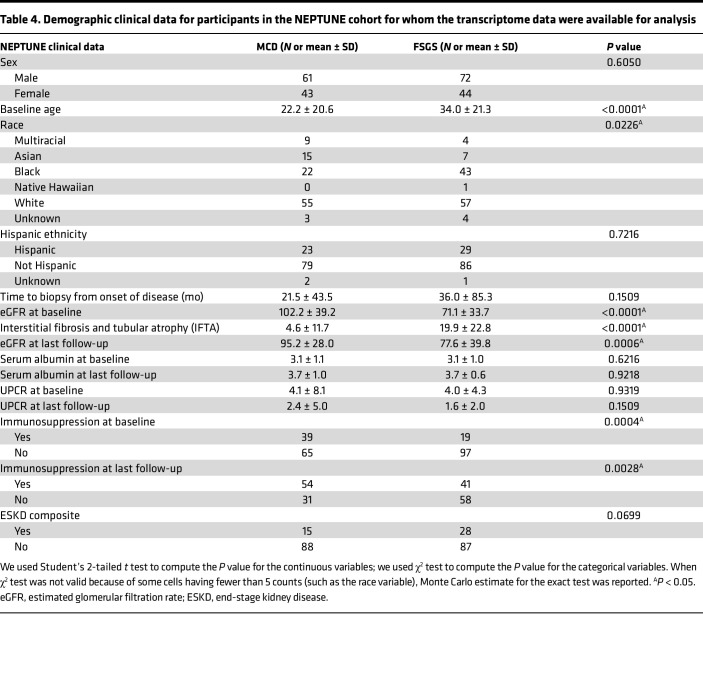
Demographic clinical data for participants in the NEPTUNE cohort for whom the transcriptome data were available for analysis

**Table 5:**
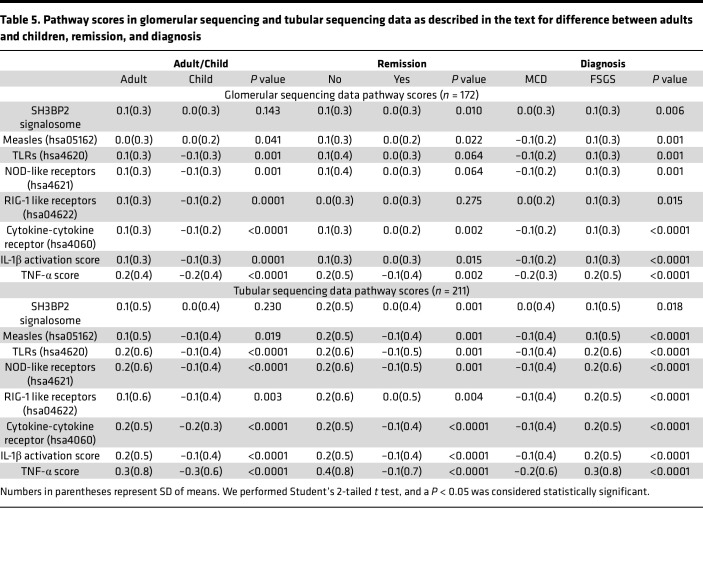
Pathway scores in glomerular sequencing and tubular sequencing data as described in the text for difference between adults and children, remission, and diagnosis

**Table 7:**
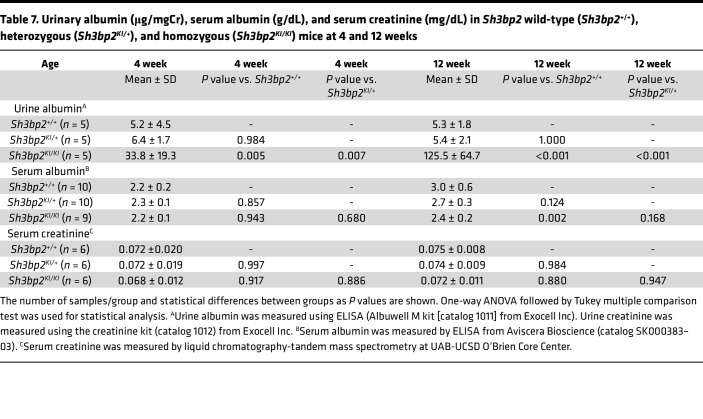
Urinary albumin (μg/mgCr), serum albumin (g/dL), and serum creatinine (mg/dL) in *Sh3bp2* wild-type (*Sh3bp2*^+/+^), heterozygous (*Sh3bp2^KI/+^*), and homozygous (*Sh3bp2^KI/KI^*) mice at 4 and 12 weeks

**Table 6:**
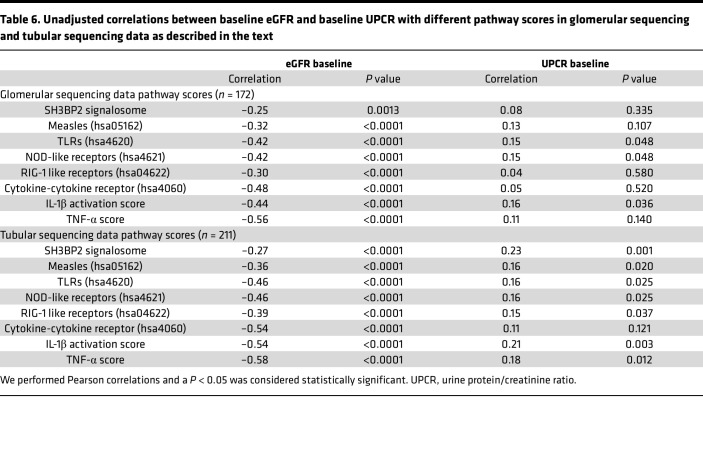
Unadjusted correlations between baseline eGFR and baseline UPCR with different pathway scores in glomerular sequencing and tubular sequencing data as described in the text

**Table 9:**
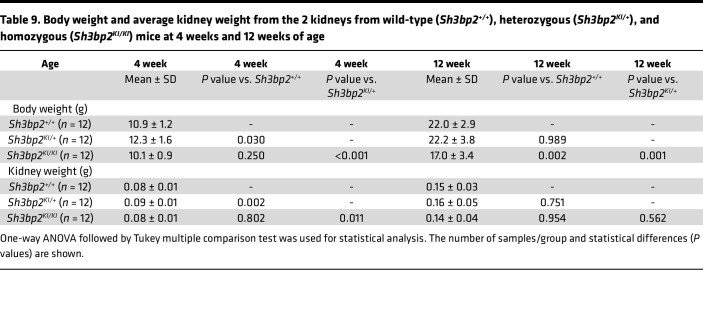
Body weight and average kidney weight from the 2 kidneys from wild-type (*Sh3bp2^+/+^*), heterozygous (*Sh3bp2^KI/+^*), and homozygous (*Sh3bp2^KI/KI^*) mice at 4 weeks and 12 weeks of age

**Table 8:**
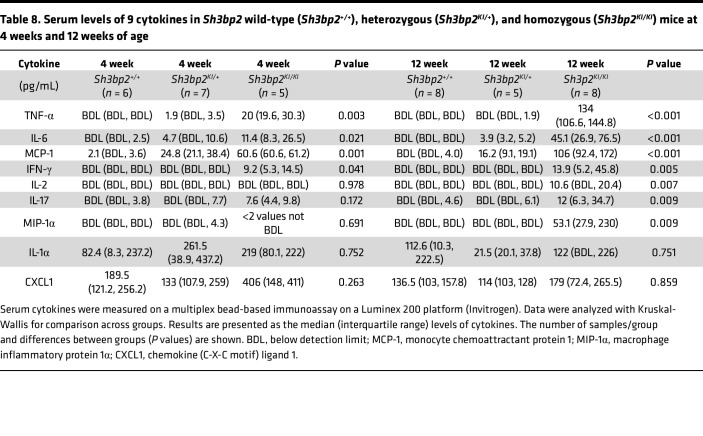
Serum levels of 9 cytokines in *Sh3bp2* wild-type (*Sh3bp2^+/+^*), heterozygous (*Sh3bp2^KI/+^*), and homozygous (*Sh3bp2^KI/KI^*) mice at 4 weeks and 12 weeks of age

**Table 3:**
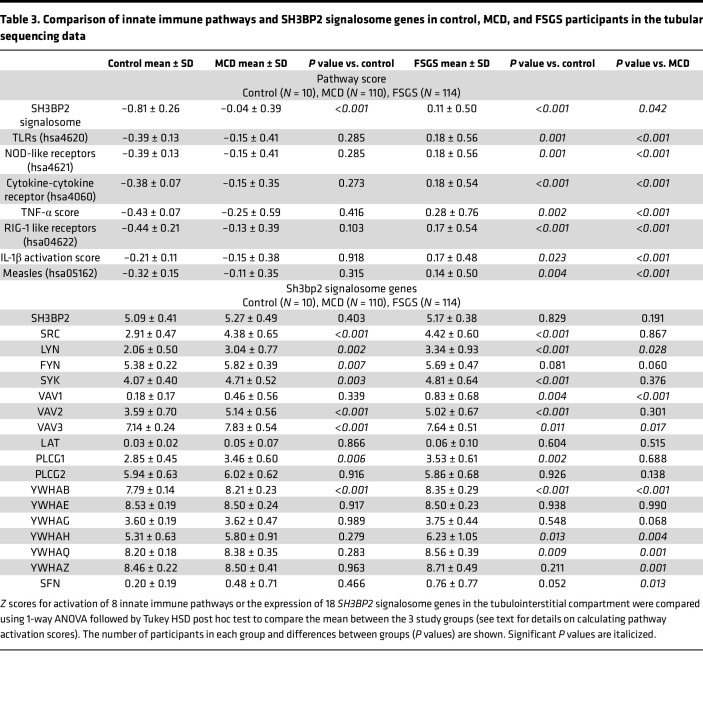
Comparison of innate immune pathways and SH3BP2 signalosome genes in control, MCD, and FSGS participants in the tubular sequencing data

**Table 2:**
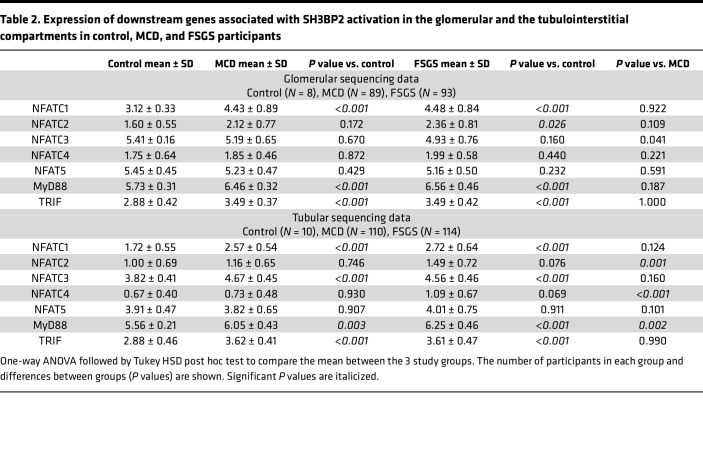
Expression of downstream genes associated with SH3BP2 activation in the glomerular and the tubulointerstitial compartments in control, MCD, and FSGS participants

**Table 1:**
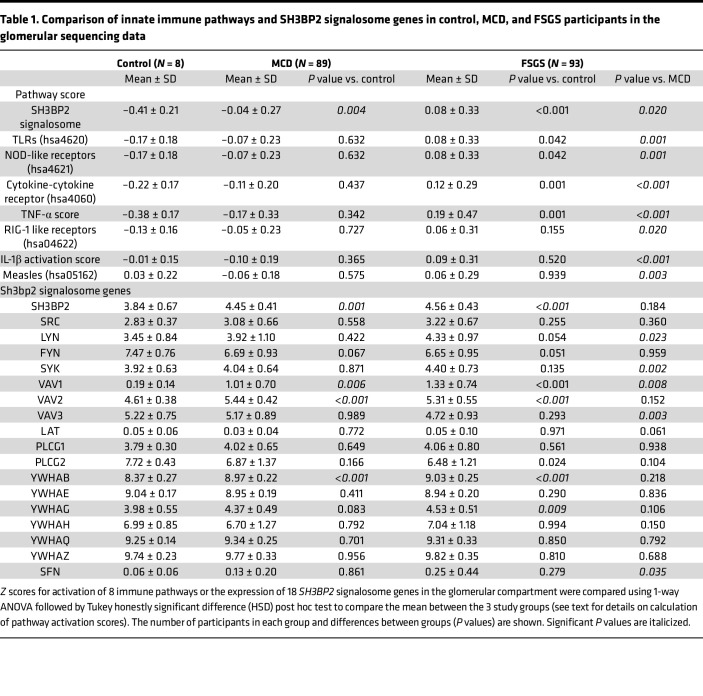
Comparison of innate immune pathways and SH3BP2 signalosome genes in control, MCD, and FSGS participants in the glomerular sequencing data

## Version 1 12/21/2023

In-Press Preview

## Version 2 02/08/2024

Electronic publication
