# Supplementary material for: Scaffold protein SH3BP2 signalosome is pivotal for immune activation in nephrotic syndrome
Source: JCI Insight. 2024 Feb 8;9(3):e170055. doi: 10.1172/jci.insight.170055 (PMC10967477; doi:10.1172/jci.insight.170055)
Supplement: Supplemental data [file jciinsight-9-170055-s167.pdf]

Supplementary Material

This PDF file includes:

1. Supplemental Material and Methods

S1A: Analysis of transcriptomic data from the NEPTUNE Study

S1B: Analysis of clinical data from the NEPTUNE Study

S1C: IMPRes analysis of transcriptomic data from the NEPTUNE Study

S1D: *Sh3bp2* Transgenic mice and samples

S1E: Urine and serum albumin and creatinine

S1F: Multiplex bead-based immunoassay for specific serum cytokines

S1G: Kidney histology by light, immunofluorescence and electron microscopy in Sh3bp2 animals

S1H: Human and mouse podocyte and mouse mesangial cell cultures

S1I: Immunoprecipitation and Western blotting

S1J: Statistical Analyses

2. Supplemental Tables

Supplemental Table S1. Demographic clinical data for (A) Children and (B) Adults for subjects with transcriptome data within the NEPTUNE cohort.

Supplemental Table S2. Survival analysis performed for each pathway.

Supplemental Table S3. Principal component analysis 1 and 2 for composite ESKD and days to ESKD, and the covariates adjusted include sex, age at baseline, ethnicity, and diagnosis, baseline eGFR, baseline UPCR, baseline and prior medication use (RAAS and immunosuppression) and IFTA score.

Supplemental Table S4. Genes used to generate the TNF activation score.

Supplemental Table S5. Genes used to generate the IL-1β activation score.

3. Supplemental Figures

Supplemental Figure S1: Pathway network map generated by the seed gene SH3BP2 in the tubulointerstitial compartment in NEPTUNE subjects.

Supplemental Figure S2: The regression tree algorithm for outcomes of ESKD composite and Remission using the eight pathway scores.

Supplemental Figure S3: SH3BP2 gene is expressed in both the glomerular and tubulointerstitial compartments of the kidney, and is detected across various kidney cell types in the single-cell sequencing dataset in the Kidney Precision Medicine Project.

4. Supplemental Acknowledgments

Supplemental Material and Methods

S1A: Analysis of transcriptomic data from the NEPTUNE Study

The NEPTUNE is an ongoing prospective observational study of children and adults with primary proteinuric kidney diseases (clinicaltrials.gov NCT1209000) [1]. In NEPTUNE, a single core of kidney biopsy tissue was collected for research purposes and preserved in RNAlater and submitted to NEPTUNE laboratories at the University of Michigan for molecular analyses. Renal biopsies were micro-dissected into glomerular and tubulointerstitial compartments. mRNA was prepared from samples using the Clontech SMARTseq Ultralow v4 kit. Multiplex amplification was used to prepare cDNA with a paired end read length of 150 bases using an Illumina HiSeq4000. RNA sequencing was performed by the University of Michigan Advanced Genomics Core (https://brcf.medicine.umich.edu/cores/advanced-genomics/). Quality of the sequencing data was assessed using the FastQC tool (http://www.bioinformatics.babraham.ac.uk/projects/fastqc/). Read counts were extracted from the fastq files using HTSeq (version 0.11). [2-4]. Read counts were voom normalized (mean-variance modelling at the observational level), and batch correction was performed [5]. In brief, voom performs the following specific calculations. The counts are converted to log2-counts per million (logCPM) values, adding 0.5 to all the counts to avoid taking the logarithm of zero. The matrix of logCPM values is then optionally normalized. The lmFit function is used to fit row-wise linear models. The lowess function is then used to fit a trend to the square-root-standard-deviations as a function of an average log-count measure. The trend line is then used to predict the variance of each logCPM value as a function of its fitted value on the count scale, and the inverse variances become the estimated precision weights. Present analysis of the NEPTUNE dataset was restricted to RNA sequencing data from glomerular and tubulointerstitial compartments from biopsy proven MCD or FSGS subjects.

For aggregate pathway activation scores, a set of genes causally linked downstream of the gene or involved in the corresponding pathways were curated from literature for Sh3bp2, IL-1β and TNFα scores or were downloaded from the KEGG database [3,4]. The KEGG database was curated for pathways known to be associated with innate immunity and identified hsa04060 (Cytokine-cytokine receptor interaction), hsa04620 (Toll-like receptor signaling pathway), hsa04621 (NOD-like receptor signaling pathway), hsa04622 (RIG-I-like receptor signaling pathway), and hsa05162 (Measles) pathways with 295, 104, 184, 70, and 139 genes, respectively. A list of genes associated with IL-1β (inflammasome) and TNFα score curated from literature is provided in Supplemental Tables S1 and S2.

The gene expression values for pathway genes were Z-transformed. Z-scores were calculated according to the definition of z-score: for each gene, the original expression was subtracted from the mean of all samples and divided by the standard deviation. A z-score was generated for each gene for each patient. The individual z-scores across all the genes in the pathway were averaged to calculate the composite pathway activation score as previously described for JAK-STAT and TNFα scores [6,7].

S1B: Analysis of clinical data from the NEPTUNE Study

NEPTUNE demographic and clinical variables were used in the analysis. Diagnosis of MCD or FSGS was confirmed by the NEPTUNE central pathologists. For the analysis, immunosuppression, and renin angiotensin aldosterone blockade (RAASi) were each coded as a binary variable with 0 indicating no immunosuppression or RAASi before or at the time of kidney biopsy, and 1 where concurrent or prior immunosuppression or RAASi was reported, respectively. Remission was coded as a binary variable with 0 indicating not in remission both before baseline or since baseline, and 1 indicating in remission either before baseline or since baseline. Urine protein/creatinine ratio (UPCR) was reported as a unitless (mg/mg) continuous

variable from NEPTUNE baseline visit. Estimated glomerular filtration rate (eGFR) was calculated using the CKiD bedside equation for children under 18 years of age, CKD-Epi equation for adults 26 years and older, and the average of the two equations for ages 18 to 26 years. Interstitial fibrosis (IFTA) in the kidney biopsy was scored by the NEPTUNE pathologists and recorded as the percentage of interstitial fibrosis and it was recorded as a continuous variable. End stage kidney disease (ESKD) was defined as onset of dialysis, transplant or eGFR < 15 ml/min/1.73m2. ESKD composite outcome was defined as ESKD or 40% loss of eGFR (and eGFR < 90).

S1C: IMPRes analysis of transcriptomic data from the NEPTUNE Study

For analysis using the IMPRes algorithm, a background pathway network was constructed using pathway information in KEGG complemented by integrating the Protein-Protein Interaction data. Starting from one or more seed genes, IMPRes explores the paths in a stepwise manner using a dynamic programming algorithm with a customized penalty function [8,9]. Seed genes are defined based on prior knowledge and target genes are defined based on differentially expressed genes. Then, the nodes, edges and pathways in the background network are assigned penalties based on expression data. The active pathway problem is formulated as a shortest path optimization problem, and the final active pathways are detected by truncating and backtracking. Thus, the strength of IMPRes method lies in a new stepwise active pathway detection using dynamic programming to develop *in silico* hypotheses.

S1D: *Sh3bp2* Transgenic mice and samples

Animal studies were approved by the Institutional Animal Care and Use Committee at the VA Medical Center, Kansas City, MO. Mice were maintained at AAALAC-approved facility with unrestricted access to food and water under light/dark cycle of 12/12 hours. The study was carried out in compliance with the ARRIVE guidelines. *Sh3bp2*$^{KI/KI}$ mice have a proline-to-

arginine (P416R) substitution in exon 9 of murine *Sh3bp2* gene on the C57BL6/J background. Heterozygous *Sh3bp2*$^{KI/+}$ mice were used for breeding to obtain *Sh3bp2*$^{KI/KI}$ animals. Genotyping for wild-type and mutant mice were performed on mouse tails using PCR primers:

5'-CTTGGAGACTGGGCTTAAGAGGAC-3'

5'-TAATACTGGTAAGCAGGGGTGCTG-3'

for the Sh3bp2 gene in wild-type, heterozygous and homozygous mutants.

Kidneys from 4-week-old and 12-week-old *Sh3bp2*$^{KI/KI}$ (homozygous), *Sh3bp2*$^{KI/+}$ (heterozygous) and *Sh3bp2*$^{+/+}$ (wildtype) mice were harvested for analysis. Parts of the kidney tissue were fixed in 10% formalin for paraffin embedding, embedded in optimal cutting temperature compound (OCT), or fixed in glutaraldehyde for electron microscopy. Blood was collected by cardiac puncture at the time of euthanasia. Urine was collected on the day prior to harvesting of kidneys.

S1E: Urine and serum albumin and creatinine

Urine albumin was measured using an Albuwell M kit (#1011, Exocell Inc, Philadelphia, PA, USA) and urine creatinine using a companion creatinine kit (#1012, Exocell Inc) following the manufacturer's instructions. Serum albumin was measured using ELISA kit from Aviscera Bioscience, Santa Clara, CA, USA (SK000383-03) following the manufacturer's instructions. Serum creatinine was measured using LC-MS/MS at UAB/UCSD O'Brien Core Center [10].

S1F: Multiplex bead-based immunoassay for specific serum cytokines

Cytokines (TNF-α, IL-6, MCP-1, IL-2, IFN-γ, IL-17, MIP-1α, IL-1α and CXCL1) in mouse serum were assayed using a Luminex 200TM platform (Invitrogen). Serum (50μl) or standards of known protein concentrations (duplicates) were added to antibody-conjugated beads. Biotinylated antibodies and streptavidin-conjugated R-phycoerythrin were used for detection.

Cytokine concentration was determined by extrapolating sample fluorescence signal to the standard curve.

S1G: Kidney histology by light, immunofluorescence and electron microscopy in Sh3bp2 animals

Paraffin embedded tissues were sectioned at 3-5μm, stained for Jones methenamine silver stain and evaluated for glomerular characteristics i.e., mesangial matrix and mesangial cellularity using an Olympus BX60 microscope (Hamburg, Germany). A scale of 0 to 3 was used to perform semi-quantitative analysis of 50 consecutive glomeruli observed from one end of the kidney section to the other. Each glomerulus examined was assigned a score as follows: 0=0-3, 1=4-5, 2=6-7 and 3=>8 cells/mesangial stalk area.

Glutaraldehyde-fixed tissue was used for electron microscopy using JEOL 1400 Plus transmission electron microscope. Photomontages of images at 5000, 15000 and 25000 magnifications were evaluated for changes in endothelial cells, mesangial cells, and podocytes, and for the presence of electron dense deposits. Briefly, quantitative analysis for the number of filtration slits per glomerular basement membrane length was performed by selecting >75 % filtering surface of 4-6 linear segments of capillary loops from each glomerulus (5 glomeruli at 25,000x) from *Sh3bp2*$^{+/+}$, *Sh3bp2*$^{KI/+}$ and *Sh3bp2*$^{KI/KI}$ animals (n=3 each group) [11]. The mean of each measurement was used to obtain the number of filtration slits per length of the glomerular basement membrane.

Protein expression for podocalyxin, PLCγ2 and Vav2 was evaluated in mouse kidney by immunofluorescence using antibodies for goat anti-mouse podocalyxin (R&D Systems, Minneapolis, MN, AF1556, 1:500 dilution), mouse anti-mouse PLCγ2 (Mouse IgG, R&D Systems, Minneapolis, MN, MAB3716, 1:100 dilution), and mouse anti-mouse VAV2 (Santa

Cruz Biotechnology Inc, Santa Cruz, CA, sc-271442, 1:100 dilution) with secondary antibodies Alexa Fluor plus 488 (1:500 dilution) Donkey anti-goat (Catalog #A32758) and Alexa Fluor plus 594 (1:200 dilution) Goat anti-mouse (Catalog #A11005) from Invitrogen, Waltham, MA, USA.

S1H: Human and mouse podocyte and mouse mesangial cell cultures

Human podocytes: Conditionally immortalized human podocytes (kind gift from Moin Saleem, Bristol Children's Hospital, Bristol, UK) with a thermosensitive variant of SV-40 as a transgene were used [12]. Cells were cultured in RPMI-1640 with insulin (10ug/ml), transferrin (5.5ug/ml), selenium (5ng/ml Na selenite), fetal bovine serum (10% v/v), 100 U/ml penicillin and 0.1 mg/ml streptomycin (Invitrogen, Carlsbad, CA) at 33°C under humidified 95% air and 5% CO_2 with change of the medium every 2 days. Podocytes were thermo-switched from 33°C to 37°C at 50-60% confluency. At 33°C under permissive conditions the T-antigen expression is active and podocytes proliferate. At 37°C under non-permissive conditions the T-antigen is inactivated and podocytes become growth-arrested within 2-3 days, start to differentiate indicated by expression of podocyte specific proteins. The differentiated podocyte phenotype is reached between 8-12 days and used on day 14.

Mouse podocytes: Conditionally immortalized mouse podocyte line (kind gift from Peter Mundel, Harvard Medical School, Boston, USA) with thermosensitive tsA58 mutant T antigen was used in these studies. Podocytes were propagated in RPMI 1640 with L-glutamine supplemented with 10% fetal bovine serum, 100 U/ml penicillin, and 0.1 mg/ml streptomycin (Invitrogen, Carlsbad, CA) under permissive conditions (33°C with 10 U/ml of γ-interferon, Cell Sciences, Norwood, MA). To induce differentiation, cells were transferred to nonpermissive conditions (37°C without γ-interferon). The differentiated podocyte phenotype is reached between 8-12 days and used on day 14.

Mouse mesangial cells: Mouse mesangial cells (ATCC CRL-1927) were grown in complete growth medium containing a 3:1 mixture of DMEM and Ham’s F12 medium (ATCC) with 14mM

HEPES (Gibco) and supplemented with 5% FBS. Cells were grown at 37°C and used at approximately 80% confluency.

S1I: Western blotting and Immunoprecipitation

Cultured podocytes were lysed in RIPA buffer containing protease and phosphatase inhibitors. Total protein was quantitated using a bicinchoninic acid protein assay kit (BCA1, Sigma-Aldrich, St. Louis, MO). Immunoprecipitation was carried out by mixing 2-5 μg antibodies Sh3bp2 (mouse monoclonal, Santa Cruz Biotechnology Inc, Santa Cruz, CA, sc-377020), PLCγ2 (mouse monoclonal, R&D Systems, Minneapolis, MN, MAB3716), and VAV2 (mouse monoclonal, Santa Cruz Biotechnology Inc, Santa Cruz, CA, sc-271442) with 75 μL/mL of Protein G Sepharose beads (2 mg/mL, P3296, Sigma-Aldrich, St. Louis, MO, USA) for 1 h. Centrifuged beads were washed and incubated with 200 μg aliquot of protein lysate overnight at 4°C. Protein bound agarose beads were washed with PBS and resuspended in sample buffer and 20 μL and used for Western blotting as described previously [13-15]. Briefly, proteins were denatured in the sample buffer containing β-mercaptoethanol at 94°C for 5 min. For other Western blots, total protein (10–20 μg/lane) was electrophoresed in 10% Tris–Glycine gel using SDS-PAGE and transferred to a PVDF membrane, washed with TBST (0.1% Tween-20), and blocked using 5% non-fat milk powder. TBST-washed membrane was incubated with HRP-conjugated secondary antibody to each primary antibody. Chemiluminescence reagent (Pierce, Rockford, IL) was added and chemiluminescence was detected using iBright FL100 imaging system (Invitrogen, Waltham, MA, USA).

S1J: Statistical Analyses

Data with normal distribution were analyzed using one-way ANOVA followed by Tukey Multiple Comparison Test for 3 group comparisons, non-normal distribution was analyzed across the

groups using Kruskal-Wallis test, and Student’s t-test for 2 group comparisons using the SPSS 24 statistical software (IBM, Armonk, NY, USA). A p value <0.05 was considered significant.

In the NEPTUNE clinical data analysis, the three outcomes of interest included ESKD composite (0/1), remission (0/1) and the number of days to ESKD. The covariates included in the regression analysis included diagnosis, sex, age at baseline, child *vs.* adult, RAASi, immunosuppression, baseline UPCR, baseline eGFR, and interstitial fibrosis. T-test was used to examine differences in pathway scores of subgroups, i.e., children *vs*. adult, remission status (yes *vs.* no), and diagnosis (FSGS *vs.* MCD). Pearson correlations were calculated between each pathway score and the baseline values of eGFR and UPCR, respectively. Univariate logistic regression and survival analysis were performed to examine if each of the pathway scores was significantly associated with ESKD composite and a survival outcome (ESKD days), adjusting for covariates. Due to the high correlations among the eight pathway scores, principal component analysis (PCA) was carried out as a dimension reduction tool. The goal of PCA was to explain the total variance of pathway scores by a fewer number of orthogonal principal components (PCs). The number of PCs selected was based on the total variance explained and the PCs were then used as the predictors in models. The analyses were carried out using SAS (version 9.4, Cary, NC: SAS Institute Inc) and R packages rpart (version 4.1.16) and rpart.plot (version 3.1.0).

References:

1. Gadegbeku CA, Gipson DS, Holzman LB, Ojo AO, Song PX, Barisoni L, Sampson MG, Kopp JB, Lemley KV, Nelson PJ, Lienczewski CC, Adler SG, Appel GB, Cattran DC, Choi MJ, Contreras G, Dell KM, Fervenza FC, Gibson KL, Greenbaum LA, Hernandez JD, Hewitt SM, Hingorani SR, Hladunewich M, Hogan MC, Hogan SL, Kaskel FJ, Lieske JC, Meyers KE, Nachman PH, Nast CC, Neu AM, Reich HN, Sedor JR, Sethna CB, Trachtman H, Tuttle KR, Zhdanova O, Zilleruelo GE, Kretzler M. Design of the Nephrotic Syndrome Study Network (NEPTUNE) to evaluate primary glomerular nephropathy by a multidisciplinary approach. Kidney Int. 2013 Apr;83(4):749-56. doi: 10.1038/ki.2012.428. PMID: 23325076; PMCID: PMC3612359.
2. Mariani LH, Eddy S, AlAkwaa FM, McCown PJ, Harder JL, Nair V, Eichinger F, Martini S, Ademola AD, Boima V, Reich HN, El Saghir J, Godfrey B, Ju W, Tanner EC, Vega-Warner V, Wys NL, Adler SG, Appel GB, Athavale A, Atkinson MA, Bagnasco SM, Barisoni L, Brown E, Cattran DC, Coppock GM, Dell KM, Derebail VK, Fervenza FC, Fornoni A, Gadegbeku CA, Gibson KL, Greenbaum LA, Hingorani SR, Hladunewich MA, Hodgin JB, Hogan MC, Holzman LB, Jefferson JA, Kaskel FJ, Kopp JB, Lafayette RA, Lemley KV, Lieske JC, Lin JJ, Menon R, Meyers KE, Nachman PH, Nast CC, O'Shaughnessy MM, Otto EA, Reidy KJ, Sambandam KK, Sedor JR, Sethna CB, Singer P, Srivastava T, Tran CL, Tuttle KR, Vento SM, Wang CS, Ojo AO, Adu D, Gipson DS, Trachtman H, Kretzler M. Precision nephrology identified tumor necrosis factor activation variability in minimal change disease and focal segmental glomerulosclerosis. Kidney Int. 2023 Mar;103(3):565-579. doi: 10.1016/j.kint.2022.10.023. PMID: 36442540; PMCID: PMC10347421.
3. Kanehisa M, Goto S. KEGG: kyoto encyclopedia of genes and genomes. Nucleic Acids Res. 2000 Jan 1;28(1):27-30. doi: 10.1093/nar/28.1.27. PMID: 10592173; PMCID: PMC102409.

4. Kanehisa M. Toward understanding the origin and evolution of cellular organisms. Protein Sci. 2019 Nov;28(11):1947-1951. doi: 10.1002/pro.3715. Epub 2019 Sep 9. PMID: 31441146; PMCID: PMC6798127.
5. Law CW, Chen Y, Shi W, Smyth GK. voom: Precision weights unlock linear model analysis tools for RNA-seq read counts. Genome Biol. 2014 Feb 3;15(2):R29. doi: 10.1186/gb-2014-15-2-r29. PMID: 24485249; PMCID: PMC4053721.
6. Tao J, Mariani L, Eddy S, Maecker H, Kambham N, Mehta K, Hartman J, Wang W, Kretzler M, Lafayette RA: JAK-STAT signaling is activated in the kidney and peripheral blood cells of patients with focal segmental glomerulosclerosis. Kidney Int, 94: 795-808, 2018 10.1016/j.kint.2018.05.022.
7. Tao J, Mariani L, Eddy S, Maecker H, Kambham N, Mehta K, Hartman J, Wang W, Kretzler M, Lafayette RA: JAK-STAT Activity in Peripheral Blood Cells and Kidney Tissue in IgA Nephropathy. Clin J Am Soc Nephrol, 15: 973-982, 2020 10.2215/cjn.110109192.
8. Jiang Y, Liang Y, Wang D, Xu D, Joshi T. A dynamic programing approach to integrate gene expression data and network information for pathway model generation. Bioinformatics. 2020 Jan 1;36(1):169-176. doi: 10.1093/bioinformatics/btz467. PMID: 31168616.
9. Jiang Y, Wang D, Xu D, Joshi T. IMPRes-Pro: A high dimensional multiomics integration method for *in silico* hypothesis generation. Methods. 2020 Feb 15;173:16-23. doi: 10.1016/j.ymeth.2019.06.013. Epub 2019 Jun 17. PMID: 31220603.
10. Young S, Struys E, Wood T. Quantification of creatine and guanidinoacetate using GC-MS and LC-MS/MS for the detection of cerebral creatine deficiency syndromes. Curr Protoc Hum Genet. 2007 Jul;Chapter 17:Unit 17.3. doi: 10.1002/0471142905.hg1703s54. PMID: 18428409.
11. Deegens JK, Dijkman HB, Borm GF, Steenbergen EJ, van den Berg JG, Weening JJ, Wetzels JF. Podocyte foot process effacement as a diagnostic tool in focal segmental

glomerulosclerosis. Kidney Int. 2008 Dec;74(12):1568-76. doi: 10.1038/ki.2008.413. Epub 2008 Aug 27. PMID: 18813290.

12. Saleem MA, O'Hare MJ, Reiser J, Coward RJ, Inward CD, Farren T, Xing CY, Ni L, Mathieson PW, Mundel P. A conditionally immortalized human podocyte cell line demonstrating nephrin and podocin expression. J Am Soc Nephrol. 2002 Mar;13(3):630-638. doi: 10.1681/ASN.V133630. PMID: 11856766.
13. Srivastava T, McCarthy ET, Sharma R, Kats A, Carlton CG, Alon US, Cudmore PA, El-Meanawy A, Sharma M. Fluid flow shear stress upregulates prostanoid receptor EP2 but not EP4 in murine podocytes. Prostaglandins Other Lipid Mediat. 2013 Jul-Aug;104-105:49-57. doi: 10.1016/j.prostaglandins.2012.11.001. Epub 2012 Dec 20. PMID: 23262148.
14. Srivastava T, Alon US, Cudmore PA, Tarakji B, Kats A, Garola RE, Duncan RS, McCarthy ET, Sharma R, Johnson ML, Bonewald LF, El-Meanawy A, Savin VJ, Sharma M. Cyclooxygenase-2, prostaglandin E2, and prostanoid receptor EP2 in fluid flow shear stress-mediated injury in the solitary kidney. Am J Physiol Renal Physiol. 2014 Dec 15;307(12):F1323-33. doi: 10.1152/ajprenal.00335.2014. Epub 2014 Sep 18. PMID: 25234310.
15. Srivastava T, Dai H, Heruth DP, Alon US, Garola RE, Zhou J, Duncan RS, El-Meanawy A, McCarthy ET, Sharma R, Johnson ML, Savin VJ, Sharma M. Mechanotransduction signaling in podocytes from fluid flow shear stress. Am J Physiol Renal Physiol. 2018 Jan 1;314(1):F22-F34. doi: 10.1152/ajprenal.00325.2017. Epub 2017 Sep 6. PMID: 28877882; PMCID: PMC5866353.

Supplemental Table S1. Demographic clinical data for (A) Children and (B) Adults for subjects with transcriptome data (NEPTUNE cohort).

**(A) Children (<18 years of age at baseline)**	MCD (N) or Mean ± SD	FSGS (N) or Mean ± SD	P-value
SEX			0.3263
-Male	41	21	
-Female	22	17	
BASELINE AGE	8.6±4.6	11.1±4.7	0.0091*
RACE			0.0075*
-Multi-Racial	8	2	
-Asian	10	2	
-Black	12	19	
-White	31	12	
-Unknown	2	3	
Hispanic			0.7652
-Hispanic	14	11	
-Not Hispanic	47	26	
-Unknown	2	1	
Time to biopsy from onset of disease (Months)	21.7±31.8	20.3±33.8	0.8447
IFTA	0.9±1.6	6.3±8.6	0.0007*
eGFR at baseline	116.1±35.8	93.9±28.8	0.0017*
eGFR at last follow up	105.3±25.1	92.5±44.2	0.1217
Serum Albumin at baseline	3.1±1.0	2.9±1.1	0.4336
Serum Albumin at last follow up	3.5±1.1	3.6±0.8	0.6345
UPCR at baseline	4.7±10.0	4.8±4.8	0.9639
UPCR at last follow up	3.2±6.1	1.5±2.3	0.0735
Immunosuppression at baseline			0.0950
-Yes	34	14	

-No	29	24	
Immunosuppression at last follow up<br>-Yes<br>-No	 39<br>14	 25<br>11	0.6697
ESKD Composite<br>-Yes<br>-No	 9<br>53	 13<br>25	0.0210*

**[B] Adults (>18 years of age at baseline)**	MCD (N) or Mean + SD	FSGS (N) or Mean + SD	P-value
SEX<br>-Male<br>-Female	 20<br>21	 51<br>27	0.0793
BASELINE AGE	43.1+17.9	45.2+16.7	0.5346
RACE<br>-Multi-Racial<br>-Asian<br>-Black<br>-Native Hawaiian<br>-White<br>-Unknown	 1<br>5<br>10<br>0<br>24<br>1	 2<br>5<br>24<br>1<br>45<br>1	0.8277
Hispanic<br>-Hispanic<br>-Not Hispanic	 9<br>32	 18<br>60	0.8892
Time to biopsy from onset of disease (Months)	21.1+58.7	45.4+103.9	0.1596
IFTA	9.9+17.0	27.6+24.7	<0.0001
eGFR at baseline	80.2+34.1	60.4+30.5	0.0017*

eGFR at last follow up	78.1±24.2	68.9±34.4	0.1406
Serum Albumin at baseline	3.0±1.1	3.3±1.0	0.2292
Serum Albumin at last follow up	4.0±0.8	3.8±0.5	0.1286
UPCR at baseline	3.1±3.5	3.6±4.0	0.4824
UPCR at last follow up	1.2±1.7	1.6±1.8	0.2651
Immunosuppression at baseline			0.2797
-Yes	5	5	
-No	36	73	
Immunosuppression at last follow up			0.0348*
-Yes	15	16	
-No	17	47	
ESKD Composite			0.5122
-Yes	6	15	
-No	35	62	

Supplemental Table S2. Survival analysis performed for each pathway. We performed univariate logistic/survival analysis to examine if the pathway was significantly associated with the composite end point (40% loss of baseline eGFR and end stage kidney disease (ESKD)) and Days to ESKD. The covariates adjusted in the logistic/survival analysis included sex, age at baseline, whether child or not, diagnosis, baseline eGFR, baseline UPCR, baseline and prior medication use (RAAS and immunosuppression) and IFTA score.

**Glomerular Sequencing Data**	ESKD composite		Days to ESKD	
Pathway Scores (n=172)	OR (CI)	p-value	HR (CI)	p-value
SH3BP2 Signalosome	1.60(0.34,7.45)	0.551	1.47(0.38,5.68)	0.578
Measles (hsa05162)	0.99(0.15,6.62)	0.996	1.92(0.32,11.57)	0.476
Toll-like receptors (hsa4620)	0.69(0.12,3.87)	0.676	1.30(0.24,6.89)	0.761
NOD-like receptors (hsa4621)	0.69(0.12,3.87)	0.676	1.30(0.24,6.89)	0.761
RIG-1 like receptors (hsa04622)	0.52(0.09,3.20)	0.484	0.65(0.14,3.03)	0.579
Cytokine-Cytokine receptor (hsa4060)	1.68(0.22,12.93)	0.619	6.10(0.83,44.90)	0.076
IL-1β activation score	2.25(0.31,16.23)	0.423	4.21(0.66,26.74)	0.128
TNFα score	1.51(0.41,5.54)	0.531	2.16(0.63,7.39)	0.219

**Tubular Sequencing Data**	ESKD composite		Days to ESKD	
Pathway Scores (n=211)	OR (CI)	p-value	HR (CI)	p-value
SH3BP2 Signalosome	0.52(0.18,1.48)	0.219	0.54(0.23,1.25)	0.150
Measles (hsa05162)	0.68(0.24,1.93)	0.467	0.63(0.28,1.43)	0.264
Toll-like receptors (hsa4620)	0.72(0.27,1.86)	0.493	0.66(0.31,1.41)	0.281
NOD-like receptors (hsa4621)	0.72(0.27,1.86)	0.493	0.66(0.31,1.41)	0.281
RIG-1 like receptors (hsa04622)	0.62(0.23,1.66)	0.338	0.58(0.25,1.31)	0.189
Cytokine-Cytokine receptor (hsa4060)	0.98(0.30,3.19)	0.971	0.78(0.29,2.08)	0.617
IL-1β activation score	0.65(0.20,2.15)	0.481	0.62(0.23,1.70)	0.356
TNFα score	0.96(0.43,2.12)	0.912	0.86(0.43,1.72)	0.674

Supplemental Table S3. Principal component analysis 1 and 2 for ESKD composite and Days to ESKD, and the covariates adjusted include sex, age at baseline, whether child or not, diagnosis, baseline eGFR, baseline UPCR, baseline and prior medication use (RAAS and immunosuppression) and IFTA score.

Glomerular sequencing	ESKD composite		Days to ESKD	
n=172	OR (CI)	p-value	HR (CI)	p-value
Principal 1	1.02(0.82,1.27)	0.85	1.13(0.91,1.39)	0.27
Principal 2	1.52(0.88,2.61)	0.13	1.49(0.94,2.35)	0.09
Age	1.01(0.97,1.05)	0.70	0.99(0.96,1.03)	0.75
Child vs adult	3.62(0.53,24.7)	0.19	2.54(0.46,13.99)	0.28
MCD vs FSGS	0.69(0.22,2.13)	0.52	1.20(0.45,3.18)	0.71
Male vs Female	1.31(0.50,3.46)	0.58	1.42(0.62,3.23)	0.40
RAAS block	1.99(0.72,5.49)	0.18	1.65(0.69,3.97)	0.26
Immunosuppression	2.02(0.55,7.46)	0.29	1.05(0.37,3.01)	0.92
UPCR	1.02(0.96,1.09)	0.54	1.01(0.96,1.07)	0.59
eGFR	1.01(0.99, 1.03)	0.53	1.01(0.99,1.02)	0.46
IFTA	1.04(1.01, 1.08)	0.01	1.06(1.03,1.08)	<0.0001

Tubular sequencing	ESKD composite		Days to ESKD	
n=211	OR (CI)	p-value	HR (CI)	p-value
Principal 1	0.96(0.78,1.18)	0.70	0.95(0.81,1.12)	0.52
Principal 2	0.64(0.33,1.26)	0.20	0.67(0.37,1.22)	0.19
Age	1.00(0.96,1.04)	0.94	0.98(0.95,1.02)	0.38
Child vs adult	3.40(0.57,20.33)	0.18	1.60(0.36,7.16)	0.54
MCD vs FSGS	0.76(0.28,2.01)	0.58	0.93(0.42,2.04)	0.85
Male vs Female	1.80(0.72,4.49)	0.21	1.65(0.75,3.65)	0.21
RAAS block	1.42(0.57,3.54)	0.45	1.35(0.67,2.75)	0.40
Immunosuppression	2.19(0.68,7.10)	0.19	1.49(0.59,3.79)	0.40
UPCR	1.03(0.97,1.09)	0.40	1.03(0.99,1.07)	0.20
eGFR	1.01(0.99,1.03)	0.21	1.01(0.99,1.02)	0.26
IFTA	1.06(1.02,1.09)	0.0005	1.06(1.03,1.08)	<0.0001

Supplemental Table S4. Genes used to generate the TNF activation score.

Gen	PMII	Gene	PMID	Gene	PMID	Gene	PMID	Gene8	PMID	Gene1	PMID1	Gene12	PMID1
CCL17	12354417	CHUK	11796489	EMCN	11594763	ICAM1	15963988	ITGB2	12454401	OLR1	11985903	SYTL1	12137562
CCL19	10679062	COX2	10501211	ENG	17389265	ICOSLG	10744980	JUN	17500068	OPTN	10807909	TAP1	1385520
CCL2	9825772	CPNE1	14674885	EPCAM	10867614	IER3	11244505	JUNB	10903323	ORM1	9726030	TFRC	9135559
CCL20	11133838	CR1	2961377	ESAM	15505101	IGFBP1	10070049	KIT	10206577	OSM	12097485	TGFB1	15574511
CCL22	11923841	CRH	12676571	ETS1	11229456	IGFBP3	11971816	KITLG	15579452	PAPPA	16269458	THBS1	11157717
CCL27	11821900	CRHR2	17412781	F3	11058594	IGFBP6	12054123	LAMP3	15963988	PECAM1	7686548	TIMP1	15663564
CCL3	12115625	CRP	9726030	FABP2	11329616	IKBKB	11796489	LEP	12032749	PLA2G4A	10930295	TIMP2	15663564
CCL4	9570566	CSF1	3494061	FABP4	12927809	IL10	12414777	LIF	8895217	PLAT	12692009	TLR2	11160251
CCL5	9277410	CSF2	12562880	FANCG	11181053	IL11	10467228	MADCAM1	15483224	PLAU	8601416	TLR4	11160251
CCND1	12444159	CSF3	1700731	FAS	8168998	IL12B	10513808	MAP2K6	9029150	PLAUR	8601416	TNF	16365392
CCR1	9787141	CTSG	11722574	FASLG	10358159	IL13RA2	14652008	MAP3K14	11368442	PLD1	11485559	TNFAIP3	12388275
CCR3	11884459	CX3CL1	11525637	FCER2	7643018	IL15RA	12165497	MAP4K4	16461467	POSTN	15378733	TNFRSF11B	11685652
CCR4	9787141	CXADR	12571626	FGF2	10629075	IL1A	7686496	MEFV	10807793	PRTN3	11415941	TNFRSF1A	11923841
CCR5	9787141	CXCL1	12744776	FGF7	7936642	IL1B	7994029	MIPEP	1372592	PTGES	11029586	TNFRSF1B	12067756
CCR7	15963988	CXCL10	11884459	FOS	3259871	IL1R1	8120407	MMP1	12060661	PTGS2	12576525	TNFRSF21	11753679
CCRL2	15188357	CXCL11	12627325	GADD45B	12388275	IL1RN	8331299	MMP10	8349617	PTHLH	10854575	TNFRSF8	12414777
CD14	1373513	CXCL2	10502561	GDF15	9886240	IL2	12414777	MMP12	17525194	PTPRF	10905491	TNFSF10	12218071
CD1A	11238627	CXCL3	9277410	GFAP	10674496	IL21R	11986233	MMP13	12878172	PTX3	9521058	TNFSF11	15479886
CD4	1921451	CXCL5	9277410	GJA1	12827213	IL2RA	12414777	MMP14	9212749	RB1	15611081	TNFSF15	11911831
CD40	11488834	CXCL6	12744776	GNAI3	12214898	IL3RA	1825289	MMP17	12962706	RELA	9756499	TNIP2	15378733
CD55	10477692	CXCL8	9209275	GPNMB	19320736	IL6	10467228	MMP2	25358651	RPS6KA5	9873047	TP53	10213465
CD58	1354203	CXCL9	12627325	GRIA1	12460558	IL6R	11923841	MMP3	10967046	S100A11	16339570	TRAF1	11978013
CD70	9067541	CXCR4	11160334	GSS	15378733	IL7	10961889	MMP7	9461124	SAA1	1714101	TRAF2	12067756
CD80	9824486	CYCS	12149248	HBEGF	12138120	IL7R	10779425	MMP9	25358651	SELE	8695814	TRAF3	12067756
CD83	15963988	CYLD	18245814	HCK	12237848	INHBA	1417851	MUC1	10867614	SELL	12517920	TRAF4	12067756
CD86	12230943	CYP19A1	12452446	HGF	9397161	IRF1	12713595	MUC5AC	12690113	SELP	10484438	TRAF5	12067756
CDK3	15378733	DDR1	11606478	HIF1A	12808024	ITGA1	12606473	MYC	10200535	SERPINA3	9461605	TRAF6	12067756
CDK4	15611081	DEFB4A	11702237	HLA-A	1354203	ITGA4	12759453	MYLK	15681825	SERPINB2	8824249	TRPC1	12855710
CDKN1A	12795334	DUSP10	24707477	HLA-B	3455781	ITGA5	8006453	NAMPT	11241162	SERPINE1	11402043	TXN	10555039
CEBPA	10211885	E2F1	15611081	HLA-C	1354203	ITGA6	8006453	NFKB1	9756499	SLC11A2	16224057	TYMP	14573775
CFB	7512988	EDN1	10764953	HMOX1	10330231	ITGAL	10198263	NFKB2	9529131	SLC3A2	2318252	VCAM1	10484438
CFH	1690734	EFNA1	11278471	HP	9097927	ITGAM	12747235	NFKBIA	12580918	SLC7A2	11742806	WNT5A	12165812
CFLAR	12861043	EGFR	10077640	HPSE	16384929	ITGAX	1921451	NLRP3	14662828	SMAD7	10652273	XIAP	16219905
CHRM2	15671275	EGR1	1370482	HSP90B1	15378733	ITGB1	9565575	NOS2	10601128	SOD2	9114748	ZFP36	10763822

Supplemental Table S5. Genes used to generate the IL-1β activation score.

Symbol	Entrez Gene ID	Symbol	Entrez Gene ID	Symbol	Entrez Gene ID	Symbol	Entrez Gene ID
A2M	2	CYP2E1	1571	IGFALS	3483	PIGR	5284
ABCC2	1244	CYP8B1	1582	IGFBP1	3484	PLA2G2A	5320
BDKRB1	623	DEFB4A	1673	IL1RL1	9173	PLA2G4A	5321
C3	718	DEFB4A	1673	IL1RN	3557	POMC	5443
CASP10	843	DEFB4B	100289462	IL33	90865	PTGES	9536
CASR	846	DIO1	1733	IL6	3569	PTGS1	5742
CCL11	6356	EHF	26298	IRF1	3659	PTGS2	5743
CCL2	6347	ELF3	1999	JUN	3725	PTX3	5806
CCL20	6364	ELN	2006	LBP	3929	RELA	5970
CCL3	6348	FAS	355	LCN2	3934	RIPK2	8767
CCL4	6351	FGG	2266	MIA	8190	SAA2	6289
CCL5	6352	FMR1	2332	MIF	4282	SDC4	6385
CD44	960	FOS	2353	MMP1	4312	SELE	6401
CEBPB	1051	FPR2	2358	MMP13	4322	SELENOP	6414
CFTR	1080	GBP1	2633	MMP3	4314	SLC10A1	6554
CIITA	4261	GCH1	2643	MMP7	4316	SOCS3	9021
COL2A1	1280	GNPAT	8443	MMP9	4318	SOD2	6648
CRH	1392	HAS1	3036	MYLK	4638	SOX9	6662
CRP	1401	HAS2	3037	MYLK3	91807	SPP1	6696
CSF2	1437	HIF1A	3091	NFKB1	4790	TACR1	6869
CTGF	1490	HMGA1	3159	NOS1	4842	TIMP1	7076
CXCL10	3627	HNF4A	3172	NOS2	4843	TSLP	85480
CXCL2	2920	HP	3240	NR1H4	9971	TXNIP	10628
CXCL8	3576	HPRT1	3251	NR1I3	9970	VEGFC	7424
CXCR4	7852	ICAM1	3383	PI3	5266	XYLT1	64131

Supplemental Figure S1: Pathway network map generated using the seed gene SH3BP2 in the tubulointerstitial compartment in NEPTUNE subjects. The genes belonging to the top five significant pathways (color code listed in inbox) based on p-value for the pathways on IMPRes analysis are bordered with the corresponding color.

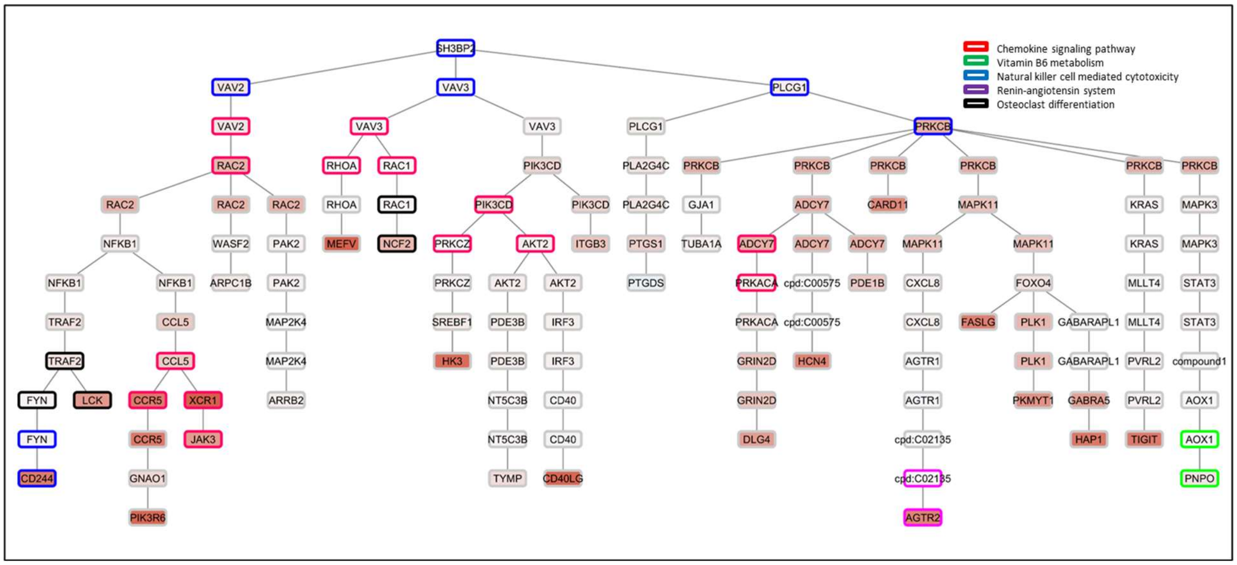

Supplemental Figure S2: We performed classification tree analysis on the pathway scores to predict outcome status for the patients for the two binary outcomes (ESKD Composite and Remission) as described in https://cran.r-project.org/web/packages/rpart/vignettes/longintro.pdf. The regression tree algorithm automatically performs from eight pathway scores for outcomes of ESKD composite and Remission. In the regression tree algorithm for each of the statements of the “tree”, if the statement is true, it will go to left side of the tree, otherwise to the right side of the tree. We predict the ESKD outcome for all the subjects with either ESKD (or Remission)="0" or ESKD (or Remission)="1". And all the "0"s are colored in blue, and all the "1"s are colored in green. The deeper the color, the higher the accuracy of the prediction. For the patients in each node of “0” or “1”, the bottom two numbers show the observed proportions of “0” and “1” values. For example, in the upper left panel (Glomerular Sequencing data), for a TNF alpha score <0.42 the algorithm predicts that those subjects do not have ESKD (ESKD=0), and in the observed data, 86% of these subjects don't have ESKD, and 14% of these subjects have ESKD, so the accuracy of this prediction is 86% correct. Similarly, for a TNF alpha score >=0.42 and toll-like receptor score <0.47, the algorithm predicts subjects to have ESKD (ESKD=1), and in the observed data, 38% don't have ESKD and 62% have ESKD, so the accuracy is 62%. Similarly we have shown regression tree algorithm for Remission in the Glomerular Sequencing data in the upper right panel, and for ESKD and Remission in the Tubulointerstitial Sequencing data in the lower panel.

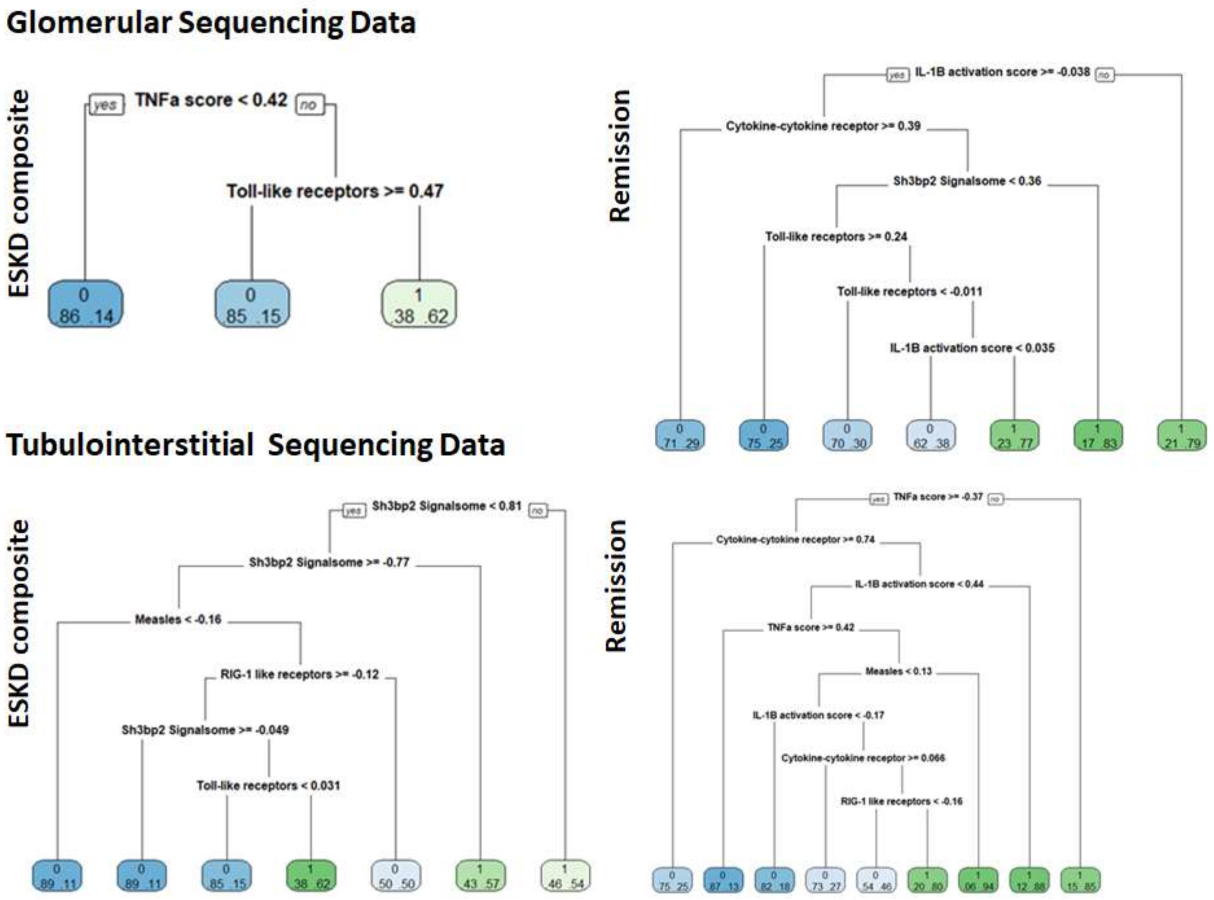

Supplemental Figure S3: Kidney Precision Medicine Project (KPMP) has single-cell sequencing dataset derived from living donor biopsies (healthy control), chronic kidney disease (CKD), and acute kidney injury (AKI). The single-cell sequencing data from the public domain website at https://atlas.kpmp.org/explorer showed SH3BP2 gene expression, while generally low, is indeed present in both the glomerular and tubulointerstitial compartments. Furthermore, SH3BP2 was detected across various kidney cell types.

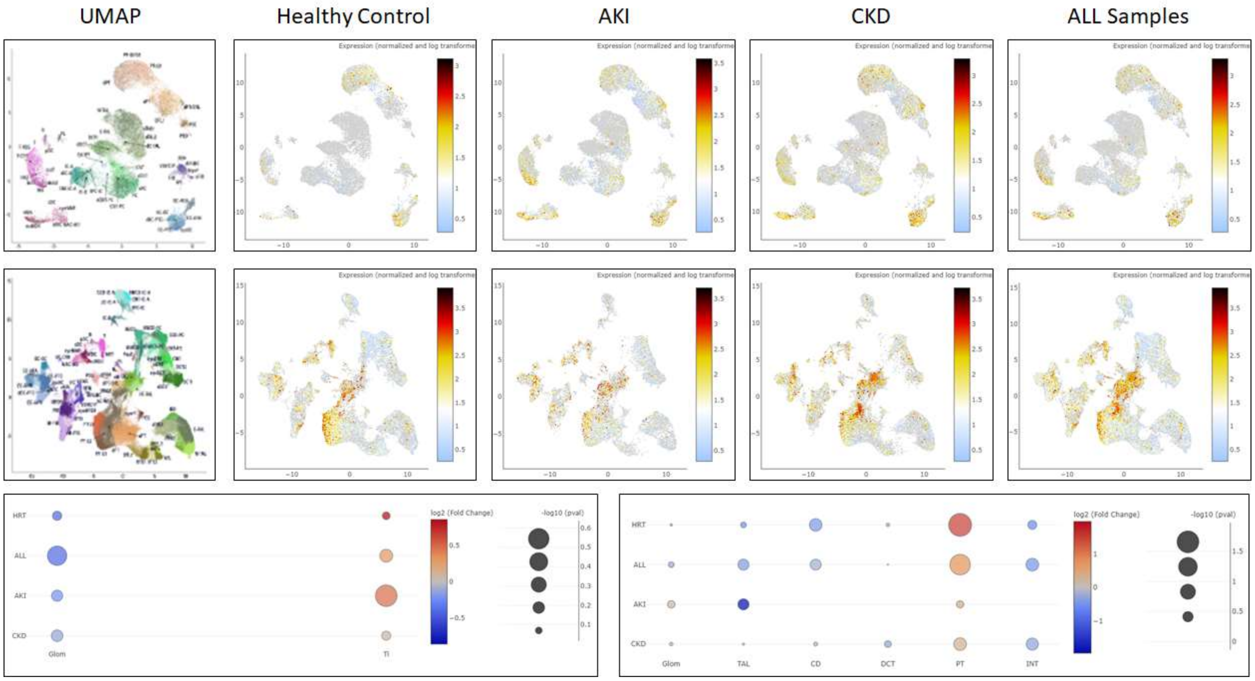

Supplemental Acknowledgments
Members of the Nephrotic Syndrome Study Network (NEPTUNE)
NEPTUNE Enrolling Centers
Cleveland Clinic, Cleveland, OH: K Dell*, J Sedor**, B Martin#
Children's Hospital, Los Angeles, CA: K Lemley*, S Tang#
Children's Mercy Hospital, Kansas City, MO: T Srivastava*, K Markus#
Cohen Children's Hospital, New Hyde Park, NY: C Sethna*, S Vento #
Columbia University, New York, NY: P Canetta*, A Pradhan#
Emory University, Atlanta, GA: L Greenbaum*, C Wang**, E Yun#
Harbor-University of California Los Angeles Medical Center: S Adler*, J LaPage#
John H. Stroger Jr. Hospital of Cook County, Chicago, IL: A Athavale*, M Itteera
Johns Hopkins Medicine, Baltimore, MD: M Atkinson*, T Dell#
Mayo Clinic, Rochester, MN: F Fervenza*, M Hogan**, J Lieske*#
Montefiore Medical Center, Bronx, NY: F Kaskel*, M Ross*, P Flynn#
NIDDK Intramural, Bethesda MD: J Kopp*
New York University Medical Center, New York, NY: L Malaga-Dieguez*, O Zhdanova**, B Pace#
Stanford University, Stanford, CA: R Lafayette*, S Dave#
Temple University, Philadelphia, PA: I Lee*, S Quinn-Boyle#
University Health Network Toronto: H Reich *, M Hladunewich**, P Ling#, M Romano#
University of Miami, Miami, FL: A Fornoni*, C Bidot#
University of Michigan, Ann Arbor, MI: M Kretzler*, D Gipson*, A Williams#, C Klida#
University of North Carolina, Chapel Hill, NC: V Derebail*, K Gibson*, A Froment#, F Ochoa-Toro#
University of Pennsylvania, Philadelphia, PA: L Holzman*, K Meyers**, K Kallem#, A Swenson#
University of Texas Southwestern, Dallas, TX: K Sambandam*, K Aleman#, M Rogers#
University of Washington, Seattle, WA: A Jefferson*, S Hingorani**, K Tuttle**§, L Manahan #, E Pao#, K Kuykendall K§
Wake Forest University Baptist Health, Winston-Salem, NC: JJ Lin*, Stefanie Baker#
Data Analysis and Coordinating Center: M Kretzler*, L Barisoni**, C Gadegbeku**, B Gillespie**, D Gipson**, L Holzman**, L Mariani**, M Sampson**, J Sedor**, J Zee**, G Alter, H Desmond, S Eddy, D Fermin, M Larkina, S Li, S Li, CC Lienczewski, T Mainieri, R Scherr, A Smith, A Szymanski, A Williams.
Digital Pathology Committee: Carmen Avila-Casado (University Health Network, Toronto), Serena Bagnasco (Johns Hopkins University), Joseph Gaut (Washington University in St Louis), Stephen Hewitt (National Cancer Institute), Jeff Hodgin (University of Michigan), Kevin Lemley (Children's Hospital of Los Angeles), Laura Mariani (University of Michigan), Matthew Palmer (University of Pennsylvania), Avi Rosenberg (Johns Hopkins University), Virginie Royal (University of Montreal), David Thomas (University of Miami), Jarcy Zee (University of Pennsylvania) Co-Chairs: Laura Barisoni (Duke University) and Cynthia Nast (Cedar Sinai).
*Principal Investigator; **Co-investigator; #Study Coordinator
§Providence Medical Research Center, Spokane, WA
